# Optimizing selection based on BLUPs or BLUEs in multiple sets of genotypes differing in their population parameters

**DOI:** 10.1007/s00122-024-04592-2

**Published:** 2024-04-15

**Authors:** Albrecht E. Melchinger, Rohan Fernando, Andreas J. Melchinger, Chris-Carolin Schön

**Affiliations:** 1 Plant Breeding, TUM School of Life Sciences, Technical University of Munich, 85354 Freising, Germany; 2 Institute of Plant Breeding, Seed Science and Population Genetics, University of Hohenheim, 70599 Stuttgart, Germany; 3 Department of Animal Science, Iowa State University, Ames, IA 50011 USA; 4 Department of Mathematics, University of Stuttgart, 70569 Stuttgart, Germany

## Abstract

**Key message:**

Selection response in truncation selection across multiple sets of candidates hinges on their post-selection proportions, which can deviate grossly from their initial proportions. For BLUPs, using a uniform threshold for all candidates maximizes the selection response, irrespective of differences in population parameters.

**Abstract:**

Plant breeding programs typically involve multiple families from either the same or different populations, varying in means, genetic variances and prediction accuracy of BLUPs or BLUEs for true genetic values (TGVs) of candidates. We extend the classical breeder's equation for truncation selection from single to multiple sets of genotypes, indicating that the expected overall selection response $({\Delta G}_{{\text{Tot}}})$ for TGVs depends on the selection response within individual sets and their post-selection proportions. For BLUEs, we show that maximizing ${\Delta G}_{{\text{Tot}}}$ requires thresholds optimally tailored for each set, contingent on their population parameters. For BLUPs, we prove that ${\Delta G}_{{\text{Tot}}}$ is maximized by applying a uniform threshold across all candidates from all sets. We provide explicit formulas for the origin of the selected candidates from different sets and show that their proportions before and after selection can differ substantially, especially for sets with inferior properties and low proportion. We discuss implications of these results for (a) optimum allocation of resources to training and prediction sets and (b) the need to counteract narrowing the genetic variation under genomic selection. For genomic selection of hybrids based on BLUPs of GCA of their parent lines, selecting distinct proportions in the two parent populations can be advantageous, if these differ substantially in the variance and/or prediction accuracy of GCA. Our study sheds light on the complex interplay of selection thresholds and population parameters for the selection response in plant breeding programs, offering insights into the effective resource management and prudent application of genomic selection for improved crop development.

**Supplementary Information:**

The online version contains supplementary material available at 10.1007/s00122-024-04592-2.

## Introduction

Selection is one of the major drivers of evolution and breeding. In nature, various types of selection occur, which are studied in evolutionary biology and described in textbooks on population genetics (e.g., Hartl et al. 1997). In breeding, directional selection is by far the most important type of selection in the sense that breeders typically select only a certain number or proportion of top candidates for a single trait or an index of the most important traits. The selected candidates are then advanced for further breeding or utilized as experimental cultivars for commercial purposes.

Cochran (1951) derived the primary mathematical results for the changes in population parameters under truncation selection in a seminal paper and demonstrated its application to plant selection. He described the selection response for a target variable, when selection is based on correlated variates. Cochran’s formula and its extension to the peculiarities in plant breeding, such as length of the breeding cycle and parental control, are known as the breeders’ equation (cf. Bernardo 2002; Lynch and Walsh 1998). This equation is one of the most important contributions of quantitative genetics to practical breeding as it quantifies the relevant factors that determine the progress expected from directional selection. However, the breeders’ equation strictly applies only to selection in a single population and assumes homogeneous correlation between the true genetic value (TGV) and the selection criterion (SC) for all candidates, which is generally not met in practice. More general settings, dropping the latter assumption, were investigated by Bulmer (1980).

In animal breeding, the problem of heterogeneity of variances among sets was early addressed in the context of different environmental groups (Brotherstone and Hill 1986). Hill (1984) found that under more intense selection, more animals are selected from the group with larger variance and recommended to correct for heterogeneity. For selection based on BLUPs, Garrick and Van Vleck (1987) examined the case of heterogeneous variances and showed that selection assuming homogeneity is still highly efficient if the prediction accuracy is high.

Plant breeding programs typically involve multiple sets of candidates from various families or populations (e.g., Auinger et al. 2021; Lian et al. 2014) and breeders often apply the same threshold to all candidates without considering their origin. However, if the sets differ in their mean and/or genetic variance and/or heritability (${h}^{2}$) of entry means, calculated as best linear unbiased estimates (BLUEs) in phenotypic selection, this may be suboptimal for the selection response of the entire program. This problem arises for example when one set of candidates is tested in more locations and/or years than another set, resulting in different heritabilities (${h}^{2}$).

When selection is based on best linear unbiased predictors (BLUPs) calculated from pedigree or “omics” data, there are numerous cases in which candidates differ in their population parameters, most notably the prediction accuracy (*ρ*) for the TGVs. In genomic selection, *ρ* strongly depends on the size of the training set and its relationship to the prediction set (e.g., Auinger et al. 2021; Clark et al. 2012; Habier et al. 2007). As demonstrated by experimental studies and simulations, adding more half-sibs to full-sibs in the training set improves *ρ* for genomic prediction within full-sib families (Brauner et al. 2019; Lehermeier et al. 2014; Lian et al. 2014; Riedelsheimer et al. 2013). Additionally, if pedigree, genomic, metabolic, or transcriptomic data are collected for different sets, the prediction accuracy of BLUPs calculated from different "omics" features or combinations of them can vary significantly among candidates (Seifert et al. 2018; Westhues et al. 2017; Zenke-Philippi et al. 2017). The same holds true for recently proposed approaches of phenomic selection based on sensor data and NIRS measurements (Robert et al. 2022; Weiß et al. 2022). Thus, breeders should be aware of the consequences of different prediction accuracies for the composition of the selected candidates.

A related, albeit slightly distinct scenario unfolds in hybrid breeding. Typically, lines from two genetically distant parent populations are selected based on predictors of their general combining ability (GCA) to attain a high selection response in the predicted hybrids (Melchinger et al. 2023). In general, breeders select an equal proportion of lines from each parent population for producing a factorial of hybrids among them (Melchinger and Posselt 2013). However, this approach may not be optimal if the two parent populations differ in their GCA variances and/or prediction accuracy for GCA effects. To our knowledge, no research has addressed the determination of the optimal proportion of lines to be selected from each parent population under this scenario.

The main objective of this study was to quantify and analyze the expected selection response when applying truncation selection to candidates from two sets differing in their population parameters. First, we extend Cochran’s formula for determining how the selection response in the combined set and the composition of the selected fraction depends on the proportion and selection response of the individual sets. Second, we derive solutions to determine the threshold, or equivalently the selected proportion, in each set to maximize the selection response in the combined set and examine the implications for selection based on BLUPs or BLUEs. Third, we explore how to optimize the selection response in hybrid breeding if the female and male parent lines of a complete factorial are selected based on their predicted GCA and the two parent populations differ in the variance and/or prediction accuracy of GCA. We augment our theoretical findings with numerical calculations that assess the benefits of utilizing optimal selected proportions and their impact on the composition of the selected set.

## Theory

The results in this section are given for two sets of genotypes ${\Pi }_{1}$ and ${\Pi }_{2}$ that can originate from the same or different populations, but they can be extended to any number of sets. The two disjoint sets ${\Pi }_{1}$ and ${\Pi }_{2}$ can be of unequal size with proportions ${\pi }_{1}$ and ${\pi }_{2}=1-{\pi }_{1}$, respectively, in the combined set ${\Pi }_{1}\cup {\Pi }_{2}$. We assume that the SC for the candidates from ${\Pi }_{1}$ or ${\Pi }_{2}$ is identically independently distributed according to normal distributions $N\left({\mu }_{1},{\sigma }_{1}^{2}\right)$ and $N\left({\mu }_{2},{\sigma }_{2}^{2}\right)$, respectively. Under these assumptions, applying truncation selection with threshold ${t}_{1}$ and ${t}_{2}$ to the candidates in ${\Pi }_{1}$ and ${\Pi }_{2}$ corresponds directly to selecting proportions $\alpha \left(\frac{{t}_{1}-{\mu }_{1}}{{\sigma }_{1}}\right)$ and $\alpha \left(\frac{{t}_{2}-{\mu }_{2}}{{\sigma }_{2}}\right)$, respectively. Here, $\alpha \left(x\right)$ denotes the proportion selected from a normal distribution $N\left(\mathrm{0,1}\right)$ using threshold $x,$ and ${i}_{\alpha \left(x\right)}$ represents the corresponding selection intensity.

In order to simplify formulas, we will use the abbreviation ${\varvec{\xi}}=\left( {\mu }_{1},{\mu }_{2},{\sigma }_{1},{\sigma }_{2},{\pi }_{1}\right)$. Thus, we get for the proportion of candidates selected from ${\Pi }_{1}\cup {\Pi }_{2}$ using thresholds ${t}_{1}$ and ${t}_{2}$ (“Appendix 1,” Eq. 17)

1 $${\alpha }_{{\text{Tot}}}({t}_{1},{t}_{2},{\varvec{\xi}})=\alpha \left(\frac{{t}_{1}-{\mu }_{1}}{{\sigma }_{1}}\right){\pi }_{1}+\alpha \left(\frac{{t}_{2}-{\mu }_{2}}{{\sigma }_{2}}\right){\pi }_{2}$$

and for the proportion of candidates from ${\Gamma }_{1}$ and ${\Gamma }_{2}$ in the selected fraction ${\Gamma }_{1}\cup {\Gamma }_{2}$ (“Appendix 1,” Eq. 18)

2 $$\gamma_{1} \left( {t_{1} ,t_{2} ,{ }{\varvec{\xi}}} \right) = \frac{{\alpha \left( {\frac{{t_{1} - \mu_{1} }}{{\sigma_{1} }}} \right)\pi_{1} }}{{\alpha_{{\text{Tot}}} \left( {t_{1} ,t_{2} ,{ }{\varvec{\xi}}} \right)}} = \frac{{\left| {\Gamma_{1} } \right|}}{{\left| {\Gamma_{1} \cup \Gamma_{2} } \right|}}\;{\text{and}}\;\gamma_{2} \left( {t_{1} ,t_{2} ,{ }{\varvec{\xi}}} \right) = \frac{{\alpha \left( {\frac{{t_{2} - \mu_{2} }}{{\sigma_{2} }}} \right)\pi_{2} }}{{\alpha_{{\text{Tot}}} \left( {t_{1} ,t_{2} ,{ }{\varvec{\xi}}} \right)}} = \frac{{\left| {\Gamma_{2} } \right|}}{{\left| {\Gamma_{1} \cup \Gamma_{2} } \right|}} = 1 - \gamma_{1} \left( {t_{1} ,t_{2} ,{ }{\varvec{\xi}}} \right)$$

Assuming the regression coefficient of the SC on the TGV is ${b}_{1}$ in ${\Pi }_{1}$ and ${b}_{2}$ in ${\Pi }_{2}$, and applying the breeders’ equation for each set, we get for the total selection response of TGVs under truncation selection in ${\Pi }_{1}\cup {\Pi }_{2}$ with thresholds ${t}_{1}$ and ${t}_{2}$ (“Appendix 1,” Eq. 22)

3 $$\begin{aligned} \Delta {G}_{{\text{Tot}}}\left({t}_{1},{t}_{2},{\varvec{\xi}},{b}_{1},{b}_{2}\right)&=\Delta {G}_{1}\left(\left({t}_{1}-{\mu }_{1}\right),{\sigma }_{1},{b}_{1}\right) {\gamma }_{1}\left({t}_{1},{t}_{2},{\varvec{\xi}}\right)+\Delta {G}_{2}\left(\left({t}_{2}-{\mu }_{2}\right),{\sigma }_{2},{b}_{2}\right){ \gamma }_{2}({t}_{1},{t}_{2},{\varvec{\xi}}) \\ & +{\mu }_{1} \left[ {\gamma }_{1}\left({t}_{1},{t}_{2},{\varvec{\xi}}\right)-{\pi }_{1}\right]+{\mu }_{2} [ {\gamma }_{2}({t}_{1},{t}_{2},{\varvec{\xi}})-{\pi }_{2}] \\ \end{aligned}$$

where $\Delta {G}_{1}\left(\left({t}_{1}-{\mu }_{1}\right),{\sigma }_{1},{b}_{1}\right)={b}_{1}{\sigma }_{1} {i}_{\alpha \left(\frac{{t}_{1}-{\mu }_{1}}{{\sigma }_{1}}\right)}$ and $\Delta {G}_{2}\left(\left({t}_{2}-{\mu }_{2}\right),{\sigma }_{2},{b}_{2}\right)={b}_{2}{\sigma }_{2} {i}_{\alpha \left(\frac{{t}_{2}-{\mu }_{2}}{{\sigma }_{2}}\right)}$ refer to the selection response in set ${\Pi }_{1}$ and ${\Pi }_{2}$, respectively.

A special situation exists in hybrid breeding with two genetically distant parent populations, where ${\Pi }_{1}$ and ${\Pi }_{2}$ correspond to sets of lines from the seed or pollen parent population, respectively. The TGV refers to the general combining ability (GCA) of each line in cross-combinations with the other parent population. Since GCA values are defined as deviations from the overall mean of the hybrid population ${\Pi }_{1}$× ${\Pi }_{2}$, we assume that the SC for the GCA of the lines from ${\Pi }_{1}$ and ${\Pi }_{2}$ follows normal distributions with $N\left(0,{\sigma }_{1}^{2}\right)$ and $N\left(0,{\sigma }_{2}^{2}\right)$, respectively, and the regression coefficients of the TGV of GCA effects on the SC are ${b}_{1}$ and ${b}_{2}$, respectively. In phenotypic selection, the SC is commonly based on the testcross performance of each line evaluated in crosses with one or several tester(s) from the opposite population of the heterotic pattern. In genomic selection, GCA can be predicted from the marker profile of the parent lines and phenotypic data of hybrids in a training set (cf. Bernardo 1996; Technow et al. 2014). The lines with highest predicted GCA effects in each parent population are generally selected for producing a factorial to be phenotyped in the final step of cultivar development (Melchinger and Posselt 2013). Thus, the selection response $\Delta {G}_{{\text{Hyb}}}$ in the complete factorial ${\Gamma }_{1}\times {\Gamma }_{2}$ of hybrids, produced by mating set ${\Gamma }_{1}$ of GCA-selected lines from ${\Pi }_{1}$ with set ${\Gamma }_{2}$ of GCA-selected lines from ${\Pi }_{2}$, compared to the factorial ${\Pi }_{1}\times {\Pi }_{2}$ among unselected lines, is equal to the sum of the selection response for GCA effects $\Delta {G}_{1}({t}_{1},{\sigma }_{1},{b}_{1})={b}_{1}{\sigma }_{1}{i}_{\alpha \left(\frac{{t}_{1}}{{\sigma }_{1}}\right)}$ plus $\Delta {G}_{2}({t}_{2},{\sigma }_{2},{b}_{2})={b}_{2}{\sigma }_{2}{i}_{\alpha \left(\frac{{t}_{2}}{{\sigma }_{2}}\right)}$ in parent population ${\Pi }_{1}$ and ${\Pi }_{2}$, respectively, and we have for ${\varvec{\theta}}$ = (${\sigma }_{1}, {\sigma }_{2}, {b}_{1},{b}_{2})$

4 $$\Delta {G}_{{\text{Hyb}}}\left({t}_{1},{t}_{2}, {\varvec{\theta}}\right)=\Delta {G}_{1}({t}_{1},{\sigma }_{1}, {b}_{1})+\Delta {G}_{2}({t}_{2},{\sigma }_{2},{b}_{2})$$

where $\alpha (\frac{{t}_{1}}{{\sigma }_{1}})=\frac{\left|{\Gamma }_{1}\right|}{\left|{\Pi }_{1}\right|}$ and $\alpha (\frac{{t}_{2}}{{\sigma }_{2}})=\frac{\left|{\Gamma }_{2}\right|}{\left|{\Pi }_{2}\right|}$ is the proportion of selected lines in ${\Pi }_{1}$ and ${\Pi }_{2}$, respectively. Note that

5 $${\alpha }_{{\text{Hyb}}}\left({t}_{1},{t}_{2},{\varvec{\theta}}\right)= \alpha \left(\frac{{t}_{1}}{{\sigma }_{1}}\right)\times \alpha \left(\frac{{t}_{2}}{{\sigma }_{2}}\right)=\frac{\left|{\Gamma }_{1}\right|}{\left|{\Pi }_{1}\right|}\times \frac{\left|{\Gamma }_{2}\right|}{\left|{\Pi }_{2}\right|}=\frac{\left|{\Gamma }_{1}\times {\Gamma }_{2}\right|}{\left|{\Pi }_{1}\times {\Pi }_{2}\right|}$$

corresponds to the proportion of hybrids in ${\Gamma }_{1}\times {\Gamma }_{2}$ selected in silico from the set of all possible hybrids in ${\Pi }_{1}\times {\Pi }_{2}$.

### Maximizing the total selection response by optimal choice of thresholds

Depending on the budget and size of the breeding program, the breeder has restrictions on the total number of genotypes to be selected from the candidates in a given cycle. This applies irrespective of whether the selected candidates are promoted to further testing for cultivar development or recombined to generate new base material for the next breeding cycle in recurrent selection. Therefore, the total proportion of selected candidates (${\alpha }_{{\text{T}}})$ is typically fixed. Nevertheless, the breeder still has the option to optimize the total selection response in ${\Pi }_{1}\cup {\Pi }_{2}$ by selecting different proportions of candidates from ${\Pi }_{1}$ and ${\Pi }_{2}$, respectively, while keeping ${\alpha }_{{\text{Tot}}}({t}_{1},{t}_{2},{\varvec{\xi}})$, the total proportion of genotypes selected from ${\Pi }_{1}\cup {\Pi }_{2}$, fixed. Thus, the goal is to find thresholds $t_{1}^{*}$ and $t_{2}^{*}$, or equivalently selected proportions ${\alpha }_{1}^{*}=\alpha \left(\frac{{t}_{1}^{*}-{\mu }_{1}}{{\sigma }_{1}}\right) \;\mathrm{and }\;{\alpha }_{2}^{*}=\alpha \left(\frac{{t}_{2}^{*}-{\mu }_{2}}{{\sigma }_{2}}\right)$, which maximize the total selection response $\Delta {G}_{{\text{Tot}}}\left({t}_{1},{t}_{2},{\varvec{\xi}},{b}_{1},{b}_{2}\right)$ under the side condition ${\alpha }_{{\text{Tot}}}\left({t}_{1},{t}_{2},{\varvec{\xi}}\right)$ = ${\alpha }_{{\text{T}}}$.

A solution to this problem can be obtained by applying a Lagrange multiplier approach. Our derivations show that $\left({t}_{1}^{*},{t}_{2}^{*}\right)$ are obtained as solutions of the following equations in $\left({t}_{1},{t}_{2}\right)$ (“Appendix 2,” Eqs. 30 and 31):

6 $$t_{1} = \frac{{b_{2} \left( {{ }t_{2} - \mu_{2} } \right) + \mu_{2} - \mu_{1} }}{{b_{1} }} + \mu_{1} \;{\text{or}}\;{\text{ equivalently}}\;t_{2} = \frac{{b_{1} \left( {t_{1} - \mu_{1} } \right) + \mu_{1} - \mu_{2} }}{{b_{2} }} + \mu_{2}$$

and

7 $${\alpha }_{{\text{Tot}}}\left({t}_{1},{t}_{2}, {\varvec{\xi}}\right)=\alpha \left(\frac{{t}_{1}-{\mu }_{1}}{{\sigma }_{1}}\right){\pi }_{1}+\alpha \left(\frac{{t}_{2}-{\mu }_{2}}{{\sigma }_{2}}\right)\left(1-{\pi }_{1}\right)= {\alpha }_{{\text{T}}}.$$

Solutions $\left({t}_{1}^{*},{t}_{2}^{*}\right)$ of these equations can be obtained by mathematical software, such as Mathematica (Wolfram 1999), and subsequently used to calculate ${\alpha }_{1}^{*}$, ${\alpha }_{2}^{*}$ and $\Delta {G}_{{\text{Tot}}}\left({t}_{1}^{*},{t}_{2}^{*},{\varvec{\xi}},{b}_{1},{b}_{2}\right)$. In order to assess the improvement in the total selection response, which can be achieved by applying optimal thresholds $\left({t}_{1}^{*},{t}_{2}^{*}\right)$ instead of identical thresholds ${t}_{1}^{i}={t}_{2}^{i}$ for both sets satisfying the side condition ${\alpha }_{{\text{Tot}}}\left({t}_{1}^{i},{t}_{2}^{i},{\varvec{\xi}}\right)={\alpha }_{{\text{T}}}$ in Eq. 7, we suggest using the ratio

8 $${\Psi }_{{\text{Tot}}}\left({\alpha }_{{\text{T}}},{\varvec{\xi}},{b}_{1},{b}_{2}\right)=100\times \left[\frac{\Delta {G}_{{\text{Tot}}}\left({t}_{1}^{*},{t}_{2}^{*}, {\varvec{\xi}},{b}_{1},{b}_{2}\right)-\Delta {G}_{{\text{Tot}}}\left({t}_{1}^{i},{t}_{2}^{i}, {\varvec{\xi}},{b}_{1},{b}_{2}\right)}{\Delta {G}_{{\text{Tot}}}\left({t}^{i},{t}^{i}, {\varvec{\xi}},{b}_{1},{b}_{2}\right)}\right].$$

In hybrid breeding, the breeder is also limited in terms of the number of promising predicted hybrids that can be evaluated in a factorial for product development in the next step of the breeding scheme. Thus, the goal is to find optimal proportions ${\alpha }_{1}^{o}$ and ${\alpha }_{2}^{o}$ of candidates from ${\Pi }_{1}$ and ${\Pi }_{2},$ or equivalently optimal thresholds ${t}_{1}^{o}$ and ${t}_{2}^{o}$, for selection in ${\Pi }_{1}$ and ${\Pi }_{2}$, respectively, which maximize the selection response $\Delta {G}_{{\text{Hyb}}}\left({t}_{1},{t}_{2},{\varvec{\theta}}\right)$ for the factorial produced between the GCA-selected lines. However, instead of using Eq. 7, the side condition takes the form

9 $${\alpha }_{{\text{Hyb}}}\left({t}_{1},{t}_{2},{\varvec{\theta}}\right)=\alpha \left(\frac{{t}_{1}}{{\sigma }_{1}}\right) \times \alpha \left(\frac{{t}_{2}}{{\sigma }_{2}}\right)={\alpha }_{{\text{H}}},$$

where ${\alpha }_{{\text{H}}}$ is the fixed proportion of hybrids to be selected for testing in the final stage of hybrid development.

A solution to this maximization problem can be found again by applying a Lagrange multiplier approach (“Appendix 3”). Accordingly, thresholds $\left({t}_{1}^{o},{t}_{2}^{o}\right)$ optimizing $\Delta {G}_{{\text{Hyb}}}\left({t}_{1},{t}_{2},{\varvec{\theta}}\right)$ in the factorial of hybrids among selected lines are found as solutions $\left({t}_{1},{t}_{2}\right)$ (“Appendix 3,” Eqs. 43) of Eq. 9 and

10 $${b}_{2}{t}_{2}-{b}_{1}{t}_{1}+\Delta {G}_{1}\left({t}_{1}{,\sigma }_{1},{b}_{1}\right)-\Delta {G}_{2}\left({t}_{2},{\sigma }_{2},{b}_{2}\right)=0.$$

Numerical solutions for $\left({t}_{1}^{o},{t}_{2}^{o}\right)$ can be obtained by mathematical software such as Mathematica and subsequently used to calculate the proportions ${\alpha }_{1}^{o}=\alpha \left(\frac{{t}_{1}^{o}}{{\sigma }_{1}}\right)$ and ${\alpha }_{2}^{o}=\alpha \left(\frac{{t}_{2}^{o}}{{\sigma }_{2}}\right)={\alpha }_{{\text{H}}}/{\alpha }_{1}^{o}$ to be selected in ${\Pi }_{1}$ and ${\Pi }_{2}$, respectively, and finally, $\Delta {G}_{{\text{Hyb}}}\left({t}_{1}^{o},{t}_{2}^{o},{\varvec{\theta}}\right)$.

In order to assess the improvement in the total selection response, which can be achieved by using the optimal proportions $\left( {\alpha_{1}^{o} ,\alpha_{2}^{o} } \right)$ compared to selecting an equal proportion ${\alpha }^{e}=\sqrt{{\alpha }_{{\text{H}}}}$ of lines from each population, i.e., using thresholds ${t}_{1}^{e}={\sigma }_{1} {\Phi }^{-1}\left(1-{\alpha }^{e}\right)$ and ${t}_{2}^{e}={\sigma }_{2}{ \Phi }^{-1}\left(1-{\alpha }^{e}\right)$, we suggest using the ratio

11 $$\Psi_{{{\text{Hyb}}}} \left( {\alpha_{{\text{H}}} ,{\varvec{\theta}}} \right) = 100 \times \left[ {\frac{{\Delta G_{{{\text{Hyb}}}} \left( {t_{1}^{o} ,t_{2}^{o} ,{ }{\varvec{\theta}}} \right) - \Delta G_{{{\text{Hyb}}}} \left( {t_{1}^{e} ,t_{2}^{e} ,{ }{\varvec{\theta}}} \right)}}{{\Delta G_{{{\text{Hyb}}}} \left( {t_{1}^{e} ,t_{2}^{e} ,{ }{\varvec{\theta}}} \right)}}} \right]{ }{\text{.}}$$

### Application to selection based on BLUPs

Let $u$ denote the random variable of true breeding values (TBVs) and $\widehat{u}$ their BLUPs, obtained by the use of pedigree or “omics” data. As shown by Henderson (1975), the standard deviation ${\sigma }_{u}$ of TGVs and the standard deviation $\sigma$ of their BLUPs are related by $\sigma =\rho {\sigma }_{u}$, where $\rho$ is the prediction accuracy, reflecting the shrinkage of BLUPs compared to the TBVs. Hence, we have ${\sigma }_{1}={\rho }_{1} {\sigma }_{{u}_{1}}$ and ${\sigma }_{2}={\rho }_{2} {\sigma }_{{u}_{2}}$. Further, the regression of $u$ on $\widehat{u}$ is equal to 1.0 for each set, so that ${b}_{1}=1.0$ and ${b}_{2}=1.0$ and this result holds true under fairly general conditions (“Appendix 4”). Thus, from Eq. 6 we obtain ${t}_{1}^{*}={t}_{2}^{*}$, even if ${\mu }_{1}\ne {\mu }_{2}$, ${\sigma }_{{u}_{1}}^{2}\ne {\sigma }_{{u}_{2}}^{2}$, and ${\rho }_{1}\ne {\rho }_{2}$. Consequently, using identical thresholds for the predicted values of TGVs (calculated as BLUPs plus the mean $\mu$ of the corresponding set) maximizes the selection response in the combined set. In conclusion, for BLUPs there is no need to search for the optimal threshold in each set and one must merely find the common threshold ${t}^{*}={t}_{1}^{*}={t}_{2}^{*}$ for both sets satisfying the side condition in Eq. 7, which can be obtained by solving the equation

12 $$\Phi \left(\frac{{t}^{*}-{\mu }_{1}}{{\sigma }_{1}}\right){\pi }_{1}+\Phi \left(\frac{{t}^{*}-{\mu }_{2}}{{\sigma }_{2}}\right)\left(1-{\pi }_{1}\right)=1-{\alpha }_{{\text{T}}}$$

Moreover, the total selection response in the combined set ${\Pi }_{1}\cup {\Pi }_{2}$ for the common threshold ${t}^{*}$ is

13 $$\begin{aligned} \Delta {G}_{{\text{Tot-BLUP}}}\left({t}^{*},{t}^{*}{\varvec{\xi}},\mathrm{1,1}\right)&=\frac{1}{{\alpha }_{{\text{T}}}}[{\sigma }_{1} \varphi \left(\frac{{t}^{*}-{\mu }_{1}}{{\sigma }_{1}}\right){\pi }_{1}+{\sigma }_{2} \varphi \left(\frac{{t}^{*}-{\mu }_{2}}{{\sigma }_{2}}\right){\pi }_{2} \\ & \quad + {\mu }_{1}{\pi }_{1}\left( \alpha \left(\frac{{t}^{*}-{\mu }_{1}}{{\sigma }_{1}}\right)-{\alpha }_{{\text{T}}}\right)+{\mu }_{2}{\pi }_{2}\left( \alpha \left(\frac{{t}^{*}-{\mu }_{2}}{{\sigma }_{2}}\right)-{\alpha }_{{\text{T}}}\right)] \\ \end{aligned}$$

### Application to selection based on BLUEs

In phenotypic selection (PS) based on BLUEs, the regression of TGVs on the SC is equal to their heritability (Falconer and Mackay 1996, p. 189), so that ${b}_{1}={h}_{1}^{2}$ and ${b}_{2}={h}_{2}^{2}$. Further, the standard deviation ${\sigma }_{u}$ of TBVs and the standard deviation σ of their BLUEs used in PS are related by $\sigma =\frac{{\sigma }_{u}}{h}$. Hence, we have ${\sigma }_{1}= {\sigma }_{{u}_{1}}/{h}_{1}$ and ${\sigma }_{2}= {\sigma }_{{u}_{2}}/{h}_{2}$. Thus, Eq. 3 becomes

14 $$\begin{aligned} \Delta G_{{\text{Tot - PS}}} \left( {t_{1} ,t_{2} , {\varvec{\xi}},h_{1}^{2} ,h_{2}^{2} } \right) & = h_{1}^{2} \sigma_{1} \varphi \left( {\frac{{t_{1} - \mu_{1} }}{{\sigma_{1} }}} \right)\pi_{1} + h_{2}^{2} \sigma_{2} \varphi \left( {\frac{{t_{2} - \mu_{2} }}{{\sigma_{2} }}} \right)\pi_{2} \\ & \quad + \mu_{1} \left[ { \gamma_{1} \left( {t_{1} ,t_{2} , {\varvec{\xi}}} \right) - \pi_{1} } \right] + \mu_{2} \left[ { \gamma_{2} \left( {t_{1} ,t_{2} , {\varvec{\xi}}} \right) - \pi_{2} } \right] \\ \end{aligned}$$

From Eqs. 6 and 7, the optimal choice of thresholds ${t}_{1}^{*}$ and ${t}_{2}^{*}$ are obtained as solutions of

15 $$\Phi \left( {\frac{{{ }t_{1}^{*} - \mu }}{{\sigma_{1} }}} \right)\pi_{1} + \Phi \left( {\frac{{h_{1}^{2} \left( {t_{1}^{*} - \mu_{1} } \right) + \mu_{1} - \mu_{2} }}{{h_{2}^{2} \sigma_{2} }}} \right)\pi_{2} = 1 - \alpha_{{\text{T}}} \;{\text{and}}\;t_{2}^{*} = \frac{{h_{1}^{2} \left( {{ }t_{1}^{*} - \mu_{1} } \right) + \mu_{1} - \mu_{2} }}{{h_{2}^{2} }} + \mu_{2} { }$$

## Numerical analyses

All equations in the theory part were programmed in software Mathematica (Wolfram 1999) for numerical analyses. As a first check for Eq. 6 and the derivations in “Appendix 2,” we numerically compared the selection response $${\Delta G}_{{\text{Tot}}}$$ for BLUPs achieved with optimized thresholds ($${t}_{1}^{*}$$, $${t}_{2}^{*})$$ versus identical ($${t}_{1}^{i}={t}_{2}^{i})$$ thresholds for BLUPs, setting $${b}_{1}={b}_{2}=1.0$$ in our program. Regardless of the means $$({\mu }_{1},$$ $${\mu }_{2})$$ and standard deviations ($${\sigma }_{1 }, {\sigma }_{2 })$$ of the SC in $${\Pi }_{1}$$ and $${\Pi }_{2}$$, as well as the choice of $${\pi }_{1}$$ and $${\alpha }_{{\text{T}}},{{\text{the value of }}}{\Delta G}_{{\text{Tot}}}$$ obtained for ($${t}_{1}^{*}$$, $${t}_{2}^{*})$$ and ($${t}_{1}^{i}={t}_{2}^{i})$$ were identical except for tiny differences attributable to rounding errors so that $${\Psi }_{{\text{Tot}}}$$ was practically zero (data not shown), confirming our theoretical results.

For BLUEs, we calculated on one hand the values for $${\Psi }_{{\text{Tot}}}$$ and $${\gamma }_{1}^{*}= { \gamma }_{1}\left({t}_{1}^{*},{t}_{2}^{*},{\varvec{\xi}}\right)$$ obtained by using the solutions for ($${t}_{1}^{*}$$, $${t}_{2}^{*})$$ obtained with the Lagrange multiplier approach (Eq. 6). On the other hand, we used Function NMaximize in Mathematica to determine the maximum of $${\Delta G}_{{\text{Tot}}}$$ under the side condition in Eq. 7. Again, the numerical results from both calculations were in perfect agreement except for numerical inaccuracies.

For finding the maximum selection response ${\Delta G}_{{\text{Hyb}}}$ in the hybrid population ${\Pi }_{1}\times {\Pi }_{2},$ we used function NMaximize in Mathematica in combination with the side condition in Eq. 9 to find the optimum choice of selected proportions$({\alpha }_{1}^{o},{\alpha }_{2}^{o})$. These values we used to calculate according to Eq. 8 the percentage improvement (${\psi }_{{\text{Hyb}} })$ in $\Delta {G}_{{\text{Hyb}}}$ when using optimized $({\alpha }_{1}^{o},{\alpha }_{2}^{o})$ instead of equal $\left({\alpha }_{1}^{e}={\alpha }_{2}^{e}\right)$ proportions of lines selected from population ${\Pi }_{1}$ and${\Pi }_{2}$.

For investigating the consequences of BLUE-based selection on the magnitude of${\Psi }_{{\text{Tot}}}$, ${\upgamma }_{1}^{*}$ and ${\gamma }_{1}^{i}= { \gamma }_{1}\left({t}_{1}^{i},{t}_{2}^{i},{\varvec{\xi}}\right)$ as a function of other relevant population parameters, we made the assumption without loss of generality that ${\mu }_{1}$ = 0 and ${\sigma }_{{u}_{1} }=1.0$. This can be achieved by centering the original SC values of all candidates as deviations from ${\mu }_{1}$ and dividing them by ${\sigma }_{1 }= {\sigma }_{{u}_{1} }/{h}_{1}$. Moreover, for representing ${\Psi }_{{\text{Tot}}}$ and ${\upgamma }_{1}^{*}$ or ${\upgamma }_{1}^{i}$ in contour plots as functions of ${\mu }_{2}$ and${h}_{2}$, we assumed ${h}_{1}^{2}=\sqrt{0.5}$ and identical genetic standard deviations in both sets (${\sigma }_{{u}_{1} }={\sigma }_{{u}_{2} })$, which closely approximates the conditions encountered in many situations in plant breeding programs.

## Software availability statement

The Mathematica programs developed for the numerical analyses of this study are available at https://github.com/TUMplantbreeding/AEM/Opt_selection_with_multiple_sets and can be downloaded from there.

## Results

**Fig. 1:**
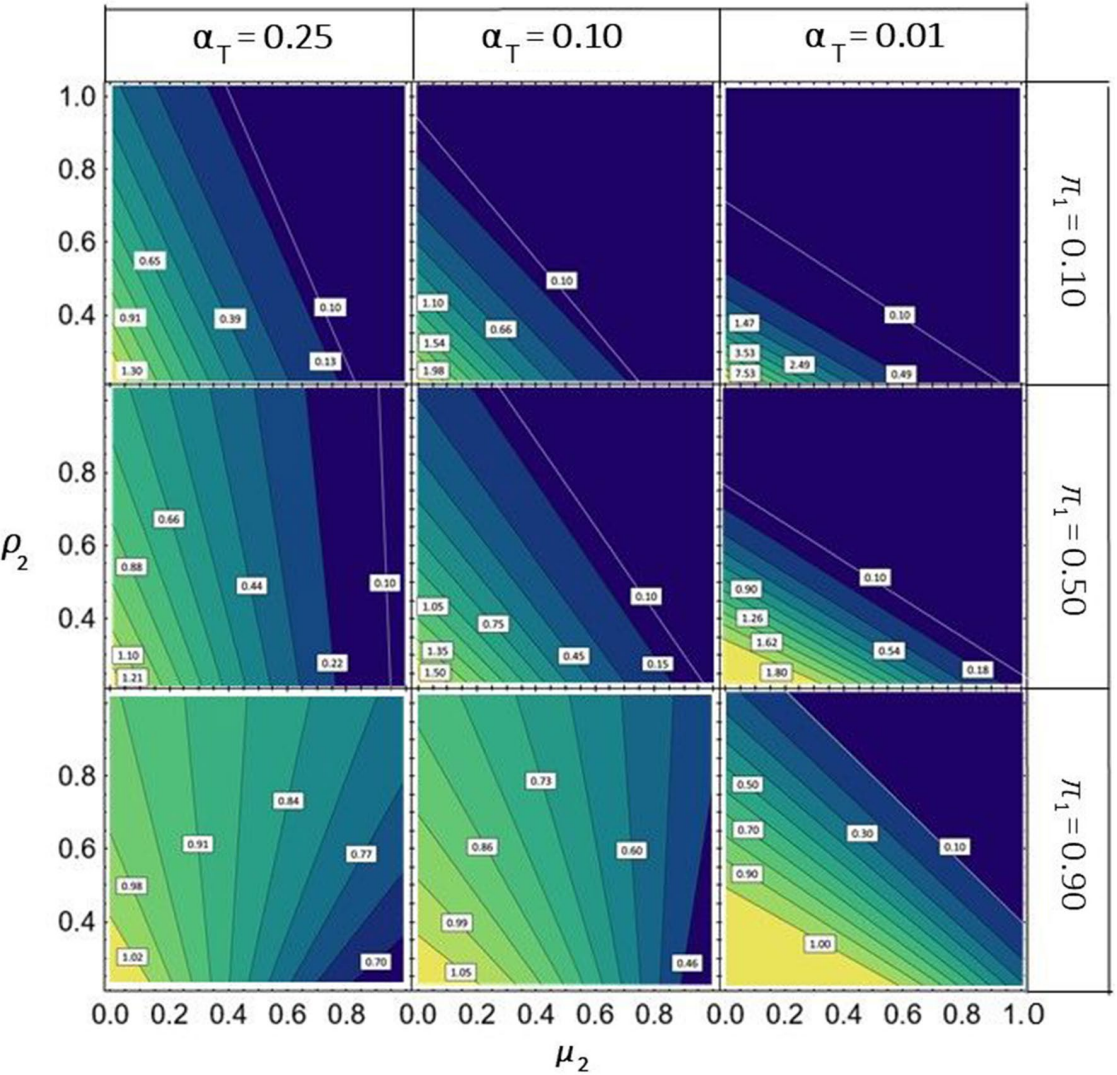
Contour plots for the ratio${\gamma }_{1}^{*}: {\pi }_{1}$, indicating the shift in the proportion of genotypes from ${\Pi }_{1}$ before (${\pi }_{1})$ and after (${\gamma }_{1}^{*})$ truncation selection based on BLUPs, when using optimal (= identical) thresholds ${(t}_{1}^{*}={t}_{2}^{*})$ in set ${\Pi }_{1}$ and${\Pi }_{2}$. The graphs show ${\gamma }_{1}^{*} :{\pi }_{1}$ as a function of the mean ${\mu }_{2}$ and the prediction accuracy ${\rho }_{2}$ of the selection criterion (SC) in ${\Pi }_{2}$ for various values of ${\pi }_{1}$ and ${\alpha }_{{\text{T}}}$, the proportion of candidates selected from ${\Pi }_{1}\cup {\Pi }_{2}$. Assumptions are ${\mu }_{1}$=0, ${\sigma }_{{u}_{1}}^{2}={\sigma }_{{u}_{2}}^{2}=1.0, {\rho }_{1}=0.50,$ i.e., ${\varvec{\xi}}$ =(0, ${\mu }_{2 },0.5, {\rho }_{2},{\pi }_{1})$. The white labels attached to the contour lines show the corresponding numerical values

Figure 1 examines for BLUPs the shift in the proportion of candidates from ${\Pi }_{1}$ before (${\pi }_{1 })$ and after selection (${\upgamma }_{1}^{*}$). We present the ratio ${\upgamma }_{1}^{*}:{\pi }_{1}$ as a function of ${\mu }_{2}\mathrm{ and }{\rho }_{2}$ under the assumptions mentioned above (${\mu }_{1}$=0,${\sigma }_{{u}_{1}}^{2}={\sigma }_{{u}_{2}}^{2}=1.0, {\rho }_{1}=0.50$). Regardless of the magnitude of ${\mathrm{\alpha }}_{{\text{T}}}$ and${\pi }_{1}$, the contour lines were straight lines, indicating that ${\upgamma }_{1}^{*}$ depends on a linear function of ${\mu }_{2}$ and ${\rho }_{2}$ with weights of these parameters determining their slope. For small values of ${\pi }_{1}$ or ${\mathrm{\alpha }}_{{\text{T}}}$, the ratio reduced substantially with an increasing sum ${\mu }_{2}$ + ${\rho }_{2}$ so that even for moderate values for one of these parameters, the ratio was smaller than 0.1, indicating that less than 10% of the initial proportion ${\pi }_{1}$ was recovered in ${\upgamma }_{1}^{*}$. For ${\pi }_{1}$= 0.90 in combination ${\mathrm{\alpha }}_{{\text{T}}}\ge 0.10$, the ratio was less affected by increasing ${\rho }_{2}$ and reduced only moderately with increasing ${\mu }_{2}$, yet the slope of the contour lines changed with ${\mu }_{2}$.

As expected, under optimal thresholds ($${t}_{1}^{*},{t}_{2}^{*})$$ for selection based on BLUEs, the contour plots for $${\upgamma }_{1}^{*}:{\pi }_{1 }$$ were identical to those obtained for BLUPs, when replacing $${\rho }_{1}$$ by $${h}_{1}= \sqrt{ {h}_{1}^{2}}$$ and $${\rho }_{2}$$ by$${h}_{2}=\sqrt{ {h}_{2}^{2}}$$, respectively (results not shown). For comparison, we also analyzed the ratio $${\upgamma }_{1}^{i} : {\pi }_{1 } \;\mathrm{as \;a \;function \;of \; }{\mu }_{2} \;\mathrm{ and}$$ $${h}_{2}$$ to monitor the relative change in the proportion of candidates from $${\Pi }_{1}$$ before ($${\pi }_{1})$$ and after selection ($${\upgamma }_{1}^{i}$$) based on BLUEs with identical thresholds ($${t}_{1}^{i}={t}_{2}^{i})$$ for both sets (Supplementary Figure 1). Compared with $${\upgamma }_{1}^{*} : {\pi }_{1}$$, the ratio $${\upgamma }_{1}^{i} : {\pi }_{1}$$ changed less with increasing $${\mu }_{2}$$ and $${h}_{2}$$, particularly for large values of $${\pi }_{1}$$ or $${\mathrm{\alpha }}_{{\text{T}}}$$. The ratio depended mainly on the magnitude of $${h}_{2}$$ and less on the size of $${\mu }_{2}$$. For $${\pi }_{1}$$ ≤ 0.50 and $${\mathrm{\alpha }}_{{\text{T}}}\ge 0.10$$, the ratio was smaller or larger than 1.0 if $${h}_{2}$$ falls below or exceeds $${h}_{1}$$, respectively, and increasing $${\mu }_{2}$$ had only a moderately reducing effect.

**Fig. 2:**
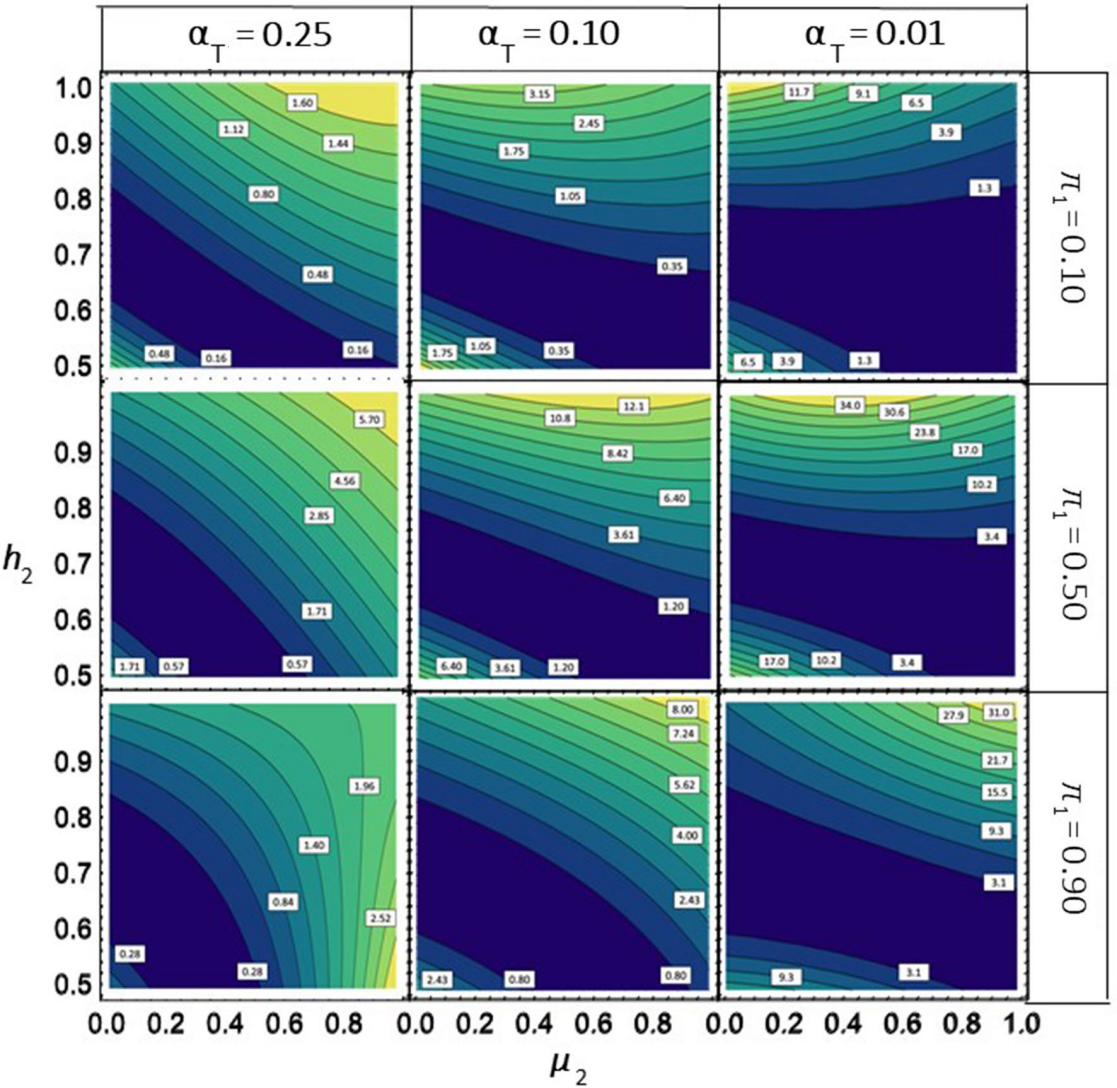
Contour plots for ${\Psi }_{{\text{Tot}}}\left({\mathrm{\alpha }}_{{\text{T}}},{\varvec{\xi}}, {h}_{1}^{2}, {h}_{2}^{2}\right)$, indicating the percentage increase of the selection response $\Delta {G}_{{\text{Tot}}}$ for selection based on BLUEs in ${\Pi }_{1}\cup {\Pi }_{2},$ when using optimal ${(t}_{1}^{*}, {t}_{2}^{*})$ versus identical ${(t}_{1}^{i}= {t}_{2}^{i})$ thresholds for truncation selection in set ${\Pi }_{1}$ and${\Pi }_{2}$, respectively. The graphs show ${\Psi }_{{\text{Tot}}}$ as a function of the mean ${\mu }_{2 }$ and ${h}_{2 },$ the square root of the heritability of the BLUEs in ${\Pi }_{2}$ for various values of ${\pi }_{1}$ and ${\alpha }_{{\text{T}}}$, the proportion of candidates selected from ${\Pi }_{1}\cup {\Pi }_{2}$. Assumptions are ${\mu }_{1}$= 0, ${\sigma }_{{u}_{1}}^{2}={\sigma }_{{u}_{2}}^{2}=1.0, {h}_{1}^{2}=0.50,$ i.e., ${\varvec{\xi}}$ = (0, ${\mu }_{2 },\sqrt{2}, \frac{1}{\sqrt{ {h}_{2}^{2}}},{\pi }_{1})$. The white labels attached to the contour lines show the corresponding numerical values

When performing mild selection ($${\mathrm{\alpha }}_{{\text{T}}}=0.25)$$ with BLUEs, the size of $${\Psi }_{{\text{Tot}}}$$, reflecting the improvement in overall selection response achieved by using optimal ($${t}_{1}^{*},{t}_{2}^{*})$$ instead of identical thresholds($${t}_{1}^{i}={t}_{2}^{i}),$$ was consistently smaller than 10%, irrespective of $${\pi }_{1 }$$ and the investigated range of $${\mu }_{2}$$ and $${h}_{2}$$ (Fig. 2). For $${\mathrm{\alpha }}_{{\text{T}}}=0.10$$, $${\Psi }_{{\text{Tot}}}$$ was close to zero for $${\pi }_{1}$$=0.1 but exceeded 10% for $${\pi }_{1}$$=0.50 and high values of $${h}_{2}$$. Under stringent selection with $${\mathrm{\alpha }}_{{\text{T}}}=0.01\; \mathrm{and }\;{h}_{2}\ge 0.90$$, $${\Psi }_{{\text{Tot}}}$$ surpassed 20% for $${\pi }_{1}$$=0.50, regardless of $${\mu }_{2},$$ or if $${\pi }_{1 }=$$ 0.50 and $${h}_{2 }\ge 0.5$$. Setting $${\mu }_{2}$$= 1.0 had only a minor effect on increasing $${\Psi }_{{\text{Tot}}}$$ compared to increasing $${h}_{2}$$ from $$\sqrt{0.5}$$ to 0.9.

**Fig. 3:**
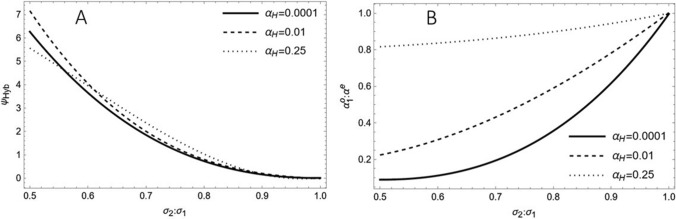
**A** Percentage increase ${\psi }_{{\text{Hyb}} }\left( {\alpha }_{{\text{H}}},{\varvec{\theta}}\right)$ of the selection response in the hybrid population ${\Pi }_{1}\times {\Pi }_{2}$ and **B** ratio of the optimal proportion of selected candidates (${\alpha }_{1}^{0})$ from ${\Pi }_{1}$ versus an equal $({\alpha }^{e}=\sqrt{ {\alpha }_{{\text{H}}}})$ proportion of lines selected from each parent population based on GCA predicted by BLUPs for ${\varvec{\theta}}=\left( {{\sigma }_{1}, {\sigma }_{2},1, 1}\right)$. The graphs show ${\psi }_{{\text{Hyb}}}$ and as function of ${\sigma }_{2}:{\sigma }_{1}$, the ratio of standard deviations of BLUPs for GCA of lines in ${\Pi }_{2}$ and ${\Pi }_{1}$, respectively, for different values of ${\alpha }_{{\text{H}}}$, the proportion of hybrids selected from ${\Pi }_{1}\times {\Pi }_{2}$

Figure 3 shows ${\Psi }_{{\text{Hyb}}},$ the increase in selection response for hybrids when selecting optimal (${\alpha }_{1}^{o},{\alpha }_{2}^{o})$ versus equal (${\alpha }_{1}={\alpha }_{2}={\alpha }^{e}=\sqrt{{\alpha }_{H }})$ proportions of lines from each parent population, as a function of ${\sigma }_{2}: {\sigma }_{1}$, the ratio of the standard deviations of BLUPs for GCA effects of lines in ${\Pi }_{1}$ and ${\Pi }_{2}$. ${\Psi }_{{\text{Hyb}}}$ showed an approximately quadratic decrease with increasing the ratio ${\sigma }_{2}: {\sigma }_{1}$ from 0.5 to 1.0 and minor differences for different values of ${\alpha }_{H }$. For ${\sigma }_{2}: {\sigma }_{1}=0.5, {\Psi }_{{\text{Hyb}}}$ was approximately 6% for all values of ${\alpha }_{H }$. The ratio ${\alpha }_{1}^{o}$: ${\alpha }^{e}$ displayed a quadratic decrease with increasing ${\sigma }_{2}: {\sigma }_{1}$ with large differences depending on ${\alpha }_{{\text{H}}}$. For ${\sigma }_{2}: {\sigma }_{1}=0.5$ and ${\alpha }_{H } \le 0.01,$ ${\alpha }_{1}^{o}$: ${\alpha }^{e}$ was smaller than 0.25, reflecting that selection of hybrids relied almost entirely on stringent GCA selection of lines in the parent population with higher variance of BLUPs and only mild selection in the other parent population.

## Discussion

### Examples of sets differing in population parameters

In all breeding categories described by Schnell (1982), plant breeders generally evaluate and select genotypes from multiple families in parallel as evident from publications on public and private breeding programs in maize and wheat (e.g., Auinger et al. 2021; Bonnett et al. 2022; Lian et al. 2014). The parents of these mostly bi-parental families generally differ in their performance level and relationship, and therefore, the progenies differ with respect to relevant population parameters. Nevertheless, these materials are routinely evaluated together in the same experiment(s) and genotypes promoted to the next stage of the program are often selected without giving much attention to their origin.

A comparable situation exists in introgression breeding programs when multiple populations are developed by crossing elite germplasm with various donors (e.g., Barbosa et al. 2021). These materials generally differ in their performance level and genetic variance due to disparate adaptation of the donors to the target environment(s) and varying proportions of donor germplasm in the pedigree. In pre-breeding programs too, the differences among populations can be extremely large as reported for landraces of maize (Böhm et al. 2017; Hölker et al. 2019; Mayer et al. 2017). If all populations are evaluated in a common experiment, breeders are inclined to apply the same threshold for identifying superior candidates used for further breeding.

Even when dealing with a single population, so that the mean and genetic variance are identical, sets of genotypes often differ with regard to the prediction accuracy of the SC for the TGV of candidates. This can be attributable to unbalanced data from multi-environment trials, where some sets are evaluated in fewer environments or replications than others. For instance, top performers remain in the testing pipeline for several years, while new entries are added to the system (Piepho et al. 2008). Moreover, some genotypes might be tested less intensively owing to problems in seed multiplication, as occurs in the production of doubled-haploid lines (Chaikam et al. 2019) or in speed breeding programs (Watson et al. 2018). Further, when complex traits are monitored using sensor-based techniques (NIRS, optical sensors, etc.) or “omics” data (genomic, phenomic, etc.), the prediction accuracy tends to be notably higher in the calibration set compared to the prediction set (Melchinger and Frisch 2023) and in sets combining different “omics” features (Schrag et al. 2018; Westhues et al. 2019). Thus, there are numerous scenarios where sets of germplasm in a breeding program differ in their population parameters and breeders should be prepared to deal adequately with these situations and be aware of the implications for selection.

### Contrasting BLUEs and BLUPs as selection criteria

Until two decades ago, selection decisions in plant breeding relied exclusively on BLUEs of the candidates, a practice that still endures in many smaller breeding programs today. Two major reasons contribute to this conservative attitude. Firstly, for traits with high heritability on an entry-mean basis, the ranking of candidates based on BLUEs and BLUPs is mostly similar. Secondly, calculation of BLUEs is straightforward and does not require information on the relationships among candidates or estimates of genetic variance components, which are challenging to obtain due to the small size of sets and rapid change over selection cycles.

Building upon the pioneering research of Henderson (1975) and inspired by the tremendous progress in animal breeding subsequent to the adoption of BLUPs, Bernardo (1994) spearheaded the implementation of BLUPs into plant breeding. With balanced data and when candidates are unrelated or possess identical co-ancestries so that their TGVs are predicted with equal accuracy, the ranking of candidates based on BLUEs and BLUPs is identical (Kennedy and Sorenson 1988). Otherwise, BLUPs offer a notable advantage by capitalizing on information from relatives and/or accommodating an efficient analysis of unbalanced data (Bernardo 2002; Piepho et al. 2008).

Another major advantage of BLUPs over BLUEs is their ability to allow direct comparisons across different breeding sets, regardless of their origin. As outlined in Eq. 6, applying the same selection threshold to the BLUPs of all candidates is optimal, whereas for BLUEs distinct thresholds must generally be found to maximize the selection response of the entire program. Following Cochran (1951), our theoretical results were derived assuming that the SC and TGVs are independently and identically distributed within each set because otherwise the already complex algebra would become even more unwieldy. This assumes an idealized situation, which is seldom met in practice as data are generally unbalanced and candidates commonly differ in their relationships. However, considering that the regression function of TGVs on BLUPs remains an identity matrix even under less stringent assumptions (“Appendix 4”), we conjecture that our results for BLUPs hold approximately true across a broad spectrum of scenarios, but this warrants further research.

**Fig. 4:**
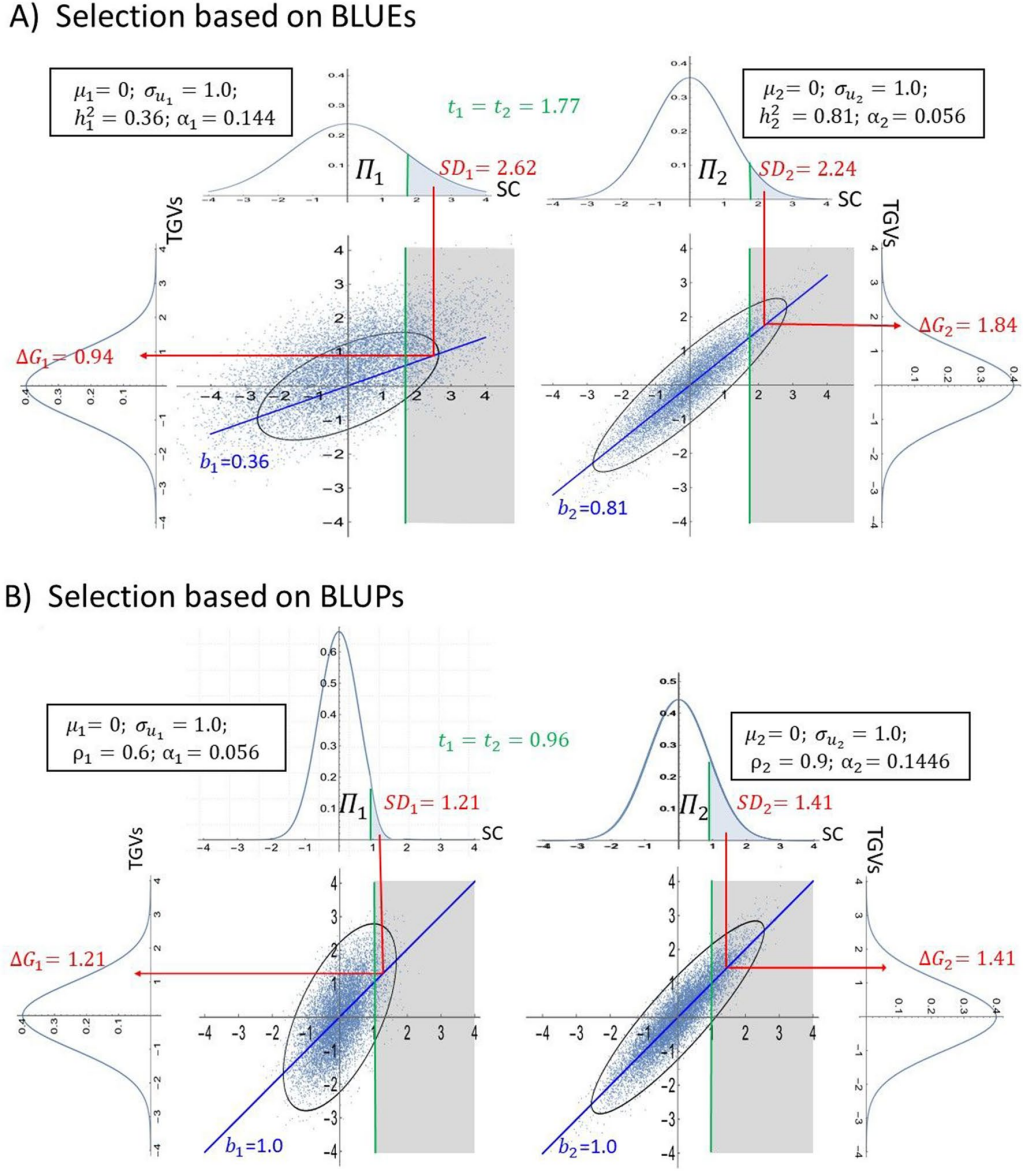
Individual and joint probability density functions (pdf) of the true genetic values (TGV ~ $N\left(\mathrm{0,1}\right)$) and selection criterion (SC ~ $N\left(0,{\sigma }^{2}\right)$ for sets ${\Pi }_{1}$ and ${\Pi }_{2}$ with equal proportions (${\pi }_{1}={\pi }_{2}=0.5) .$ $\mathbf{A} \;\mathrm{SC \;are \;BLUEs \;with }\;\sqrt{{h}_{1}^{2}}=0.6$ and $\sqrt{{h}_{2}^{2}}=0.9$ in ${\Pi }_{1}$ and ${\Pi }_{2},$ respectively. **B** SC are BLUPs with ${\rho }_{1}=0.6$ and ${\rho }_{2}=0.9$ in ${\Pi }_{1}$ and ${\Pi }_{2},$ respectively. In both cases, truncation selection with identical thresholds is practiced in ${\Pi }_{1}\cup$ ${\Pi }_{2}$ to achieve ${\alpha }_{{\text{T}}}=0.1$. SD refers to the selection differential

The difference between BLUEs and BLUPs is illustrated by two sets ${\Pi }_{1}$ and ${\Pi }_{2}$ with equal proportion (${\pi }_{1}=0.5$) of unrelated candidates sampled from the same population and selection of ${\alpha }_{{\text{T}}}=0.10$ candidates across ${\Pi }_{1}\cup {\Pi }_{2}$ (Fig. 4). Thus, the two sets share identical means (${\mu }_{1}={\mu }_{2}=0$) and genetic standard deviations (${\sigma }_{{u}_{1}}={\sigma }_{{u}_{2}}=1$). Regarding the prediction accuracy of the SC, we assume $\sqrt{{h}_{1}^{2}}={\rho }_{1}=0.6$ for ${\Pi }_{1}$ and $\sqrt{{h}_{2}^{2}}={\rho }_{2}=0.9$ for ${\Pi }_{2}$, i.e., these values differ between the two sets but are identical for BLUEs and BLUPs within each set.

When using BLUEs, the standard deviation of the SC is larger in ${\Pi }_{1}$ compared to ${\Pi }_{2}$ (${\sigma }_{1}=\frac{{\sigma }_{{u}_{1}}}{{h}_{1}}=1.67 \;{\text{vs.}}\; {\sigma }_{2}=\frac{{\sigma }_{{u}_{2}}}{{h}_{2}}=1.11$) due to the lower heritability. Utilizing identical thresholds (${t}_{1}^{i}{=t}_{2}^{i}=1.77$) for ${\alpha }_{T }=0.10$, a larger proportion of candidates is selected in ${\Pi }_{1}$ than in ${\Pi }_{2}$ (${\alpha }_{1}=0.14 \;{\text{vs.}}\; {\alpha }_{2}=0.06$), leading to lower selection intensity in ${\Pi }_{1}$ (${i}_{{\alpha }_{1}}=1.57 \;{\text{vs.}}\; {i}_{{\alpha }_{2}}=2.02$). While the selection differentials are similar (${\text{SD}}_{1}=2.62 \;{\text{vs.}}\; {\text{SD}}_{2}=2.24$), the selection response almost doubles in ${\Pi }_{2}$ compared to ${\Pi }_{1}$ ($\Delta {G}_{1}=0.94 \;{\text{vs.}}\; \Delta {G}_{2}=1.82$) owing to the higher heritability. Since the proportion of candidates selected from ${\Pi }_{1}$ is much larger than it would be with optimal thresholds ( ${\gamma }_{1}^{i}=0.72 \;{\text{vs.}}\;{\gamma }_{1}^{*}=0.28$), this explains why for BLUEs the selection response $\Delta {G}_{{\text{Tot}}}(\mathrm{1.77,1.77}, {\varvec{\xi}},\mathrm{0.36,0.81})=1.19$ is significantly smaller than the maximum selection response $\Delta {G}_{{\text{Tot}}}(\mathrm{2.65,1.18}, {\varvec{\xi}},\mathrm{0.36,0.81})=1.36$ achieved with optimal thresholds $\left({t}_{1}^{*}=2.65,{t}_{2}^{*}=1.18\right)$, resulting in ${\psi }_{{\text{Tot}}}$ = 14.5%. For very stringent selection with ${\alpha }_{{\text{T}}}$= 0.01, we get ${\gamma }_{1}^{i}=0.95.{ \;{\text{vs.}}\; \gamma }_{1}^{*}=0.05$, leading to ${\psi }_{{\text{Tot}}}$= 42.3%.

When using BLUPs as SC, candidates of ${\Pi }_{1}$ exhibit a smaller standard deviation than those of ${\Pi }_{2}$ (${\sigma }_{1}={\rho }_{1}{\sigma }_{{u}_{1}}=0.6 \;{\text{vs.}}\; {\sigma }_{2}={\rho }_{2}{\sigma }_{{u}_{2}}=0.9$) due to increased shrinkage. Consequently, applying identical thresholds (${t}_{1}^{*}{=t}_{2}^{*}=0.96$) to both sets for achieving ${\alpha }_{T }=0.10$ leads to a smaller proportion of candidates $({\alpha }_{1}=0.06 \;{\text{vs.}}\; {\alpha }_{2}= 0.14$) and a higher selection intensity $({i}_{{\alpha }_{1}}= 2.02$ vs. ${i}_{{\alpha }_{2}}=1.57$) for ${\Pi }_{1}$ compared to ${\Pi }_{2}$. Given that the regression for TGVs on BLUPs is equal to 1.0 (Henderson 1975), we obtain $\Delta {G}_{1}=1.21$ and $\Delta {G}_{2}=1.42$. Referring to Eqs. 2 and 13, we get ${\gamma }_{1}^{*}=0.28$ and ${\Delta G}_{{\text{Tot}}}(\mathrm{0.96,0.96}, {\varvec{\xi}},1, 1)=1.36$. While this example was chosen for simplicity, it underscores the fundamental disparities between BLUEs and BLUPs for selection in scenarios involving multiple sets.

### Properties of BLUPs for selection

BLUPs possess several optimality properties for prediction of random effects in mixed linear models (Fernando and Gianola 1986; Henderson 1990). They have minimum prediction error variance and maximize the correlation to the TGVs in the class of linear unbiased predictors. Furthermore, when random effects adhere to a normal distribution and fixed effects in the mixed model are known, BLUPs have smallest mean-squared error among all possible predictors. Concerning truncation selection, we provided a proof in “Appendix 2” that when dealing with two sets characterized by distinct population parameters (e.g., means, variances and prediction accuracies of TGVs), utilizing a uniform threshold for the BLUPs across all candidates maximizes the selection response.

We derived this property of BLUPs through a Lagrange multiplier approach, which requires quite restrictive assumptions on the random effects $u$ in the different sets. It is closely related to a more general selection principle (Fernando and Gianola 1986; Goffinet 1983). Accordingly, if $n$ candidates are available and $k<n$ of them are to be chosen, then selecting the $k$ candidates with highest conditional mean for an unobservable random variable $u$ maximizes the expected value of the mean of $u$ for the selected candidates. This result holds true independent on the joint distribution of the unobservable random variable $u$ and the data. Under normality, the BLUP of $u$ can be thought of as its conditional mean. Thus, even when the candidates are from different sets, selecting the $k$ candidates with the highest values for BLUPs $(\widehat{u})$ would maximize the response to selection and no further corrections are needed.

In a strict sense, selecting a fixed number $k$ or constant proportion $\alpha =$ $k/n$ of candidates from a finite population of size $n$ differs from truncation selection. In truncation selection, the threshold is set so that the expected proportion of candidates is equal to $\alpha$ in a population of infinite size. When applying this fixed threshold to a sample of size $n$, the number of selected candidates may deviate from $k$. However, as the sample size increases, selecting a fixed number or proportion of candidates becomes equivalent to truncation selection. Therefore, the results derived for truncation selection in this study closely approximate those for selecting a constant proportion of candidates.

Our approach for proving the optimality property of BLUPs under truncation selection allows calculating the optimal proportion ${\alpha }_{1}^{*}$ and ${\alpha }_{2}^{*}$ of candidates selected from set ${\Pi }_{1}$ and${\Pi }_{2}$, respectively, given reliable estimates of the population parameters are available. This information is important for optimizing the allocation of resources in genomic selection based on BLUPs. By knowing ${\alpha }_{1}^{*}$ and ${\alpha }_{2}^{*}$ in advance, we can calculate the selection response across both the training and prediction set. Thus, we can find the ideal balance between (1) the expenditures allocated to the training set, which determines mainly the prediction accuracies of both the training and prediction set, and (2) the size of both sets, which determines ${\alpha }_{{\text{T}}}$. A thorough examination of this complex problem is beyond the scope of this study and warrants further research.

Using the same threshold for BLUPs does not necessarily mean that all candidates share an equal likelihood of being selected, even if they possess the same TGV as highlighted in the literature (Woolliams et al. 2015). This can be exemplified by Fig. 4, where for ${\alpha }_{{\text{T}}}$ = 0.10 the proportion of candidates from set ${\Pi }_{1}$ would reduce from ${\pi }_{1}=$ 0.50 before selection to ${\gamma }_{1}^{*}={ \gamma }_{1}^{i}=0.28$ after selection owing to the lower prediction accuracy for ${\Pi }_{1}$ and increased shrinkage of BLUPs.

### Composition of the selected fraction

Generalizing Cochran’s formula for selection response to the case of multiple sets allowed us to examine the proportions (${\gamma }_{1}, {\gamma }_{2})$ of selected candidates originating from ${\Pi }_{1}$ and${\Pi }_{2}$. This is of interest for two reasons. First the selection response for the combined set depends on a weighted summation of the selection response in each set (Eq. 3), with weights corresponding to the post-selection fractions ${\gamma }_{1}$ and ${\gamma }_{2}$. Second, the makeup of the selected fraction is critical for further breeding progress, given that these candidates are used either directly for product development and/or for generating the base materials of the next breeding cycle. In extreme cases, ${\Delta G}_{{\text{Tot}}}$ for BLUEs can even be negative. For instance, if only mild selection ( ${\alpha }_{1}=0.45)$ is applied to the inferior, smaller population ${\Pi }_{1} ({\pi }_{1}$=0.2, ${\mu }_{1}=0$, ${\sigma }_{1}=1, {h}_{1}^{2}$= 0.36) but stringent selection (${\alpha }_{2}=0.0125)$ is applied to ${\Pi }_{2}$(${\mu }_{2}=2.0$, ${\sigma }_{2}=2$ and ${h}_{2}^{2}$= 0.81) so that ${\gamma }_{1}=0.90$ is much larger than ${\pi }_{1}$, the outcome would be${\Delta G}_{{\text{Tot}}}=-0.70$.

Here, we focus our discussion on the composition of the selected fraction obtained through the use of BLUPs with a uniform threshold for all candidates. As indicated by the graphs in Fig. 1, the change in the composition of the candidates before and after selection, expressed by the ratio ${\gamma }_{1}^{*}$: ${\pi }_{1}$, can be striking. For instance, when ${\pi }_{1}$ = 0.10 and/or ${\alpha }_{{\text{T}}}$ = 0.01, the proportion retained from the inferior set ${\Pi }_{1}$ dwindles to less than 10% of its original proportion, if ${\mu }_{2}$ surpasses ${\mu }_{1}$ by about one genetic standard deviation under otherwise identical conditions. Consequently, if materials from introgression programs are evaluated together with elite germplasm and the same threshold is applied to the BLUPs of both groups, hardly any novel germplasm will be selected due to its low performance level. Thus, it would be prudent to apply different thresholds for both groups to have a realistic chance that some of the promising new genotypes are retained for further breeding.

Likewise, the ratio${\gamma }_{1}^{*}$: ${\pi }_{1}$ falls below 0.20, if two sets share equal size and population parameters, yet ${\rho }_{2}\ge 0.68$ while ${\rho }_{1}=0.50$. Differences of this magnitude have been observed in genomic prediction of maize hybrids, in which case the prediction accuracy significantly decreased from H2 hybrids, where both parents are used as parents of a hybrid in the training set, to H1 and H0 hybrids, where only one or none of the parent lines, respectively, contribute to a hybrid in the training set (Seye et al. 2020; Technow et al. 2014; Westhues et al. 2017). While it seems rewarding to have a much larger number of H0 hybrids than H1 and H2 hybrids due to their lower costs (involving only production and genotyping of parent lines), the contribution of H0 hybrids to the overall selection response is generally overrated because their selected proportion is much smaller than for H2 and H1 hybrids owing to the lower prediction accuracy. Consequently, H0 hybrids contribute significantly less to the selection response than expected based on their proportion in the entire set of predictable hybrids. This aspect is crucial when optimizing the distribution of resources allocated to the training and prediction sets (Riedelsheimer and Melchinger 2013).

There are many further examples, where sets differ in their prediction accuracy because they differ in the number of close relatives in the training set. For this reason, genotypes in the training set have generally a significantly higher prediction accuracy than those in the prediction set, leading to a notable underrepresentation of the latter in the selected set. Likewise, in recycling breeding breeders typically generate more and larger families from crosses of elite parents. If the training set is sampled proportional to the size of these families, it follows that genotypes descending from the top parents have higher prediction accuracy due to more and closer relatives in the training set than genotypes descending from less prominent parents. Thus, on top of the expected high TGVs of these progenies, the smaller shrinkage of their BLUPs further increases the likelihood that they are selected. However, this carries a high risk of selecting closely related genotypes descending from a small number of top ancestors, thereby diminishing the effective population size and long-term progress in genomic selection, particularly when applying rigorous selection pressure enabled by the low costs for genotyping with modern methods (Rasheed et al. 2017). While our focus has been primarily on diverse prediction accuracies, our conclusions can be extended to scenarios where sets differ in genetic variances.

### Optimal selection of parent lines in hybrid breeding

In hybrid breeding, breeders typically work with a comparable number of lines from each parent population and select, based on GCA predicted by BLUPs, a proportional number of candidates from both groups for the final testing phase in product development (Melchinger and Frisch 2023). Figure 3 shows that this approach is optimal when the parent populations exhibit similar variances for the SC, but this is not always the case in practice. In European maize for example, GCA variance for grain yield was approximately twice as large for dent lines compared to flint lines (Schrag et al. 2006). Similarly in hybrid rye, Wilde et al. (2003) found that GCA variance for grain yield among female lines from the Petkus pool was almost four times greater than observed among male lines from the Carsten pool. Additionally, the accuracy of predicted GCA effects can differ between the parent populations due to differences in the size and intensity of phenotyping of the training set and the use of different types of testers. Furthermore, in species like rye, where the implementation of CMS for testcross seed production differs significantly between the seed and pollen parent pools, the pedigree relationship between candidates in the prediction and training set can diverge (Wilde and Miedaner 2021).

Under these scenarios, a notable enhancement ${\psi }_{{\text{Hyb}}}$ in selection response for predicted hybrids, compared to selecting equal proportions in each population, can be achieved by opting for more stringent selection within the parent population exhibiting the larger GCA variance. The magnitude of ${\psi }_{{\text{Hyb}}}$ depends strongly on the ratio of GCA variances in the two parent populations but showed similar curves independent of the selected proportions ${\alpha }_{{\text{H}}}$ (Fig. 3). Under mild selection (${\alpha }_{{\text{H}}}$ = 0.25), the optimal α-values for the two parent populations hardly differ from each other, but for stringent selection (${\alpha }_{{\text{H}}}$ = 0.0001), a much more stringent selection must be practiced in the parent population with larger GCA than smaller GCA variance, as reflected by the low ratio ${\alpha }_{1}^{0}$:${\alpha }^{e}$, where ${\alpha }^{e}=\sqrt{{\alpha }_{{\text{H}}}}$. As an alternative to selecting parent lines based on their predicted GCA for producing a complete factorial of hybrids, one could directly select the most promising hybrids based on the sum of the GCA of their parents. This would result in selecting a partial factorial having the form of a triangle, with the top parents being involved in more crosses than parents with lower rank and automatically takes care of differences in the GCA variance of BLUPs for each parent population. A comparison of these two selection schemes would be highly interesting for hybrid breeding but is beyond the scope of this study.

## Conclusions

When practicing truncation selection with candidates from multiple sets, new aspects must be taken into consideration as compared to selection in a single homogeneous population. This is because selection progress in the entire breeding program depends not only on the selection response in each set but also on the composition of the selected fraction. A major question is how to choose the thresholds for candidates from the various sets for maximizing the selection response of the entire breeding program. In addition to the numerous advantages of BLUPs compared to BLUEs, they have the highly desirable property that a uniform threshold can be applied to all candidates for maximizing the selection response and no further adjustment for differences in the reliability of the predictors is necessary. This applies even if the sets differ in the population parameters and/or if BLUPs of different candidates are calculated from different types or combinations of "omics" data and simplifies selection decisions. However, calculation of BLUPs requires reliable estimates of the genetic variance, which is a challenge with the small sample sizes of families used in plant breeding, but this problem has been mitigated with the use of Bayesian methods (Sorenson and Gianola 2004).

Since variation in the prediction accuracy can have a strong impact on the outcome of the selected fraction and strongly reduces the effective population size under the stringent selection, we recommend to accompany genomic selection based on BLUPs with monitoring the genetic diversity of the selected candidates. Ideally, genomic selection could be combined with optimum contribution selection (Daetwyler et al. 2007; Gaynor et al. 2021; Woolliams et al. 2015), where the relationship of candidates is determined from genomic data.

In genomic selection of hybrids based on predicted values of GCA of their parents, we suggest to select different proportions in the two parent populations, if these differ substantially in their population parameters such as the GCA variances and/or prediction accuracy of GCA effects.

### Supplementary Information

Below is the link to the electronic supplementary material.

Supplementary file1 (DOCX 342 KB)

## Appendix 1: Response to truncation selection in two sets and composition of the selected set

In our notation, we use $\varphi \left(x\right)$ and $\Phi \left(x\right)$ to denote the probability density function and cumulative distribution function of the standard normal distribution $N\left(\mathrm{0,1}\right)$, respectively; $\alpha \left(x\right)=1-\Phi \left(x\right)$ and ${i}_{\alpha (x)}$ denote the selected proportion and selection intensity, respectively, when applying threshold $x$ to a standard normal distribution $N\left(\mathrm{0,1}\right)$.

Our assumptions for the mathematical derivations are:

1. $Z$ is a Bernoulli distributed variable that indicates the origin of a candidate $C$ from set ${\Pi }_{1}$ or ${\Pi }_{2}$, where $Z=1$ with probability ${\pi }_{1}=\frac{\left|{\Pi }_{1}\right|}{\left|{\Pi }_{1}\cup {\Pi }_{2}\right|}$ for $C\in {\Pi }_{1}$ and $Z=2$ with probability ${\pi }_{2}=1-{\pi }_{1}$ for $C\in {\Pi }_{2}$.
2. $X$ is the random variable for the SC with a conditional distribution ${\left.X\right|}_{Z=1}\sim N\left({\mu }_{1},{\sigma }_{1}^{2}\right)$ for $C\in {\Pi }_{1}$ and ${\left.X\right|}_{Z=2}\sim N\left({\mu }_{2},{\sigma }_{2}^{2}\right)$ for candidates $C\in {\Pi }_{2}$.
3. Candidates $C$ from set ${\Pi }_{1}$ and ${\Pi }_{2}$ are selected based on their SC surpassing the respective thresholds ${t}_{1}$ and ${t}_{2}$, yielding the sets ${\Gamma }_{1}$ and ${\Gamma }_{2}$ of selected candidates. Choosing thresholds ${t}_{1}$ and ${t}_{2}$ is equivalent to selecting proportions ${\alpha }_{1}$ and ${\alpha }_{2}$ of top candidates from ${\Pi }_{1}$ and ${\Pi }_{2},$ respectively, where
  16 $$\begin{aligned} \alpha_{1 } & = P\left[ {\left. {X > t_{1} } \right|C \in \Pi_{1} } \right] = P\left[ {\left. X \right|_{Z = 1} > t_{1} } \right] = \left[ {1 - \Phi \left( {\frac{{t_{1} - \mu_{1} }}{{\sigma_{1} }}} \right)} \right] = \alpha \left( {\frac{{t_{1} - \mu_{1} }}{{\sigma_{1} }}} \right) \\ \alpha_{{2{ }}} & = P\left[ {\left. {X > t_{2} } \right|C \in \Pi_{2} } \right] = P\left[ {\left. X \right|_{Z = 2} > t_{2} } \right] = { }\left[ {1 - \Phi \left( {\frac{{t_{2} - \mu_{2} }}{{\sigma_{2} }}} \right)} \right] = \alpha \left( {\frac{{t_{2} - \mu_{2} }}{{\sigma_{2} }}} \right). \\ \end{aligned}$$

Our subsequent derivations are based on thresholds as dealing with proportions would further complicate the already complex algebra. Applying the theorem of total probability and defining ${\varvec{\xi}}=\left( {\mu }_{1},{\mu }_{2},{\sigma }_{1},{\sigma }_{2},{\pi }_{1},{\pi }_{2}\right)$, we obtain for the total proportion of candidates selected from${\Pi }_{1}\cup {\Pi }_{2}$:

17 $$\begin{aligned} \alpha_{{{\text{Tot}}}} \left( {t_{1} ,t_{2} ,{ }{\varvec{\xi}}} \right) & = P\left[ {\left. {X > t_{1} } \right|C \in \Pi_{1} } \right] \cdot P\left[ {C \in \Pi_{1} } \right] + P\left[ {\left. {X > t_{2} } \right|C \in \Pi_{2} } \right] \cdot P\left[ {C \in \Pi_{2} } \right] \\ { } & = P\left[ {\left. X \right|_{Z = 1} > t_{1} } \right]P\left[ {Z = 1} \right] + P\left[ {\left. X \right|_{Z = 2} > t_{2} } \right]P\left[ {Z = 2} \right]{ = }\alpha \left( {\frac{{t_{1} - \mu_{1} }}{{\sigma_{1} }}} \right)\pi_{1} + \alpha \left( {\frac{{t_{2} - \mu_{2} }}{{\sigma_{2} }}} \right)\pi_{2} \\ \end{aligned}$$

Using Bayes’ formula, the proportion of candidates from ${\Pi }_{1}$ in the entire set ${\Gamma }_{1}\cup {\Gamma }_{2}$ of selected candidates is

18 $$\gamma_{1} \left( {t_{1} ,t_{2} ,{ }{\varvec{\xi}}} \right) = \frac{{P\left[ {\left. X \right|_{Z = 1} > t_{1} } \right]P\left[ {Z = 1} \right]}}{{P\left[ {\left[ {\left. X \right|_{Z = 1} > t_{1} } \right] \vee \left[ {\left. X \right|_{Z = 2} > t_{2} } \right]} \right]}} = \frac{{\alpha \left( {\frac{{t_{1} - \mu_{1} }}{{\sigma_{{\hat{u}_{1} }} }}} \right)\pi_{1} }}{{\alpha_{{\text{Tot}}} \left( {t_{1} ,t_{2} ,{ }{\varvec{\xi}}} \right)}} = \frac{{\left| {\Gamma_{1} } \right|}}{{\left| {\Gamma_{1} \cup \Gamma_{2} } \right|}}\;{\text{and}}\;\gamma_{2} \left( {t_{1} ,t_{2} ,{ }{\varvec{\xi}}} \right) = 1 - { }\gamma_{1} \left( {t_{1} ,t_{2} ,{ }{\varvec{\xi}}} \right).$$

The expectation of the SC for the candidates in ${\Gamma }_{1}$ and ${\Gamma }_{2}$ selected from ${\Pi }_{1}$ and ${\Pi }_{2}$, respectively, is

19 $$E\left[ {\left. X \right|_{Z = 1} > t_{1} } \right] = { }\sigma_{1} { }i_{{\alpha \left( {\frac{{t_{1} - \mu_{1} }}{{\sigma_{1} }}} \right)}} + \mu_{1} \;{\text{and}} \;E\left[ {\left. X \right|_{Z = 2} > t_{2} } \right] = { }\sigma_{2} { }i_{{\alpha \left( {\frac{{t_{2} - \mu_{2} }}{{\sigma_{2} }}} \right)}} + \mu_{2} ,$$

where $\sigma {i}_{\alpha \left(\frac{t-\mu }{\sigma }\right)}$ is the selection differential under truncation selection with threshold $t$ in a normal distribution $N\left(\mu ,{\sigma }^{2}\right)$, and $i_{\alpha \left( x \right)}$ is the selection intensity (Falconer and Mackay 1996, p. 189).

Thus, we obtain the expectation of the selected candidates in ${\Gamma }_{1}\cup {\Gamma }_{2}$ as

20 $$\begin{aligned} & E\left[ {\left. X \right|\left[ {Z = 1 \wedge \left. X \right|_{Z = 1} > t_{1} } \right] \vee \left[ {\left. {Z = 2 \wedge X} \right|_{Z = 2} > t_{2} } \right]} \right] \\ & = E\left[ {\left. X \right|_{Z = 1} > t_{1} } \right]P\left[ {\left. {\left. X \right|_{Z = 1} > t_{1} } \right|\left[ {\left. X \right|_{Z = 1} > t_{1} } \right] \vee \left[ {\left. X \right|_{Z = 2} > t_{2} } \right]} \right] + E\left[ {\left. X \right|_{Z = 2} > t_{2} } \right]P\left[ {\left. {\left. X \right|_{Z = 2} > t_{2} } \right|\left[ {\left. X \right|_{Z = 1} > t_{1} } \right] \vee \left[ {\left. X \right|_{Z = 2} > t_{2} } \right]} \right] \\ & = \left[ {\sigma_{1} { }i_{{\alpha \left( {\frac{{t_{1} - \mu_{1} }}{{\sigma_{1} }}} \right)}} + \mu_{1} } \right]{ }\gamma_{1} \left( {t_{1} ,t_{2} ,{ }{\varvec{\xi}}} \right) + \left[ {\sigma_{2} { }i_{{\alpha \left( {\frac{{t_{2} - \mu_{2} }}{{\sigma_{2} }}} \right)}} + \mu_{2} } \right]{ }\gamma_{2} \left( {t_{1} ,t_{2} ,{ }{\varvec{\xi}}} \right) \\ \end{aligned}$$

Since the mean of unselected candidates in $\Pi_{1} \cup \Pi_{2}$ is obtained as 

$$E\left[ {\left. X \right|\left[ {Z = 1} \right] \vee \left[ {Z = 1} \right]} \right] = E\left[ {\left. X \right|Z = 1} \right]P\left[ {Z = 1} \right] + E\left[ {\left. X \right|Z = 2} \right]P\left[ {Z = 2} \right] = \mu_{1} \pi_{1} + \mu_{2} \pi_{2}$$

we get for the change in the mean of the SC as a result of truncation selection (= selection differential)

21 $$\begin{aligned} {\text{SD}}_{{{\text{Tot}}}} \left( {t_{1} ,t_{2} , {\varvec{\xi}}} \right) & = \sigma_{1} i_{{\alpha \left( {\frac{{t_{1} - \mu_{1} }}{{\sigma_{1} }}} \right)}} \gamma_{1} \left( {t_{1} ,t_{2} ,{ }{\varvec{\xi}}} \right){ + }\sigma_{2} i_{{\alpha \left( {\frac{{t_{2} - \mu_{2} }}{{\sigma_{2} }}} \right)}} \gamma_{2} \left( {t_{1} ,t_{2} ,{ }{\varvec{\xi}}} \right) \\ & \quad + \mu_{1} \left[ {\gamma_{1} \left( {t_{1} ,t_{2} ,{ }{\varvec{\xi}}} \right) - \pi_{1} } \right] + \mu_{2} \left[ {\gamma_{2} \left( {t_{1} ,t_{2} ,{ }{\varvec{\xi}}} \right) - \pi_{2} } \right] \\ \end{aligned}$$

Assuming the regression coefficient of the TGV on the SC is ${b}_{1}$ in ${\Pi }_{1}$ and ${b}_{2}$ in ${\Pi }_{2}$ and applying the breeders’ equation for each set, we get for the total selection response in ${\Pi }_{1}\cup {\Pi }_{2}$ under truncation selection with thresholds ${t}_{1}$ and ${t}_{2}$:

22 $$\begin{aligned} \Delta G_{{{\text{Tot}}}} \left( {t_{1} ,t_{2} ,{ }{\varvec{\xi}},b_{1} ,b_{2} } \right) & = \Delta G_{1} \left( {\left( {t_{1} - \mu_{1} } \right),\sigma_{1} ,b_{1} } \right)\gamma_{1} \left( {t_{1} ,t_{2} ,{ }{\varvec{\xi}}} \right) + \Delta G_{2} \left( {\left( {t_{2} - \mu_{2} } \right),\sigma_{2} ,b_{2} } \right)\gamma_{2} \left( {t_{1} ,t_{2} ,{ }{\varvec{\xi}}} \right) \\ & \quad + \mu_{1} \left[ {\gamma_{1} \left( {t_{1} ,t_{2} ,{ }{\varvec{\xi}}} \right) - \pi_{1} } \right] + \mu_{2} \left[ {\gamma_{2} \left( {t_{1} ,t_{2} ,{ }{\varvec{\xi}}} \right) - \pi_{2} } \right] \\ \end{aligned}$$

where $\Delta {G}_{1}\left(\left({t}_{1}-{\mu }_{1}\right),{\sigma }_{1},{b}_{1}\right)={b}_{1}{\sigma }_{1} {i}_{\alpha \left(\frac{{t}_{1}-{\mu }_{1}}{{\sigma }_{1}}\right)}$ and $\Delta {G}_{2}\left(\left({t}_{2}-{\mu }_{2}\right),{\sigma }_{2},{b}_{2}\right)={b}_{2}{\sigma }_{2} {i}_{\alpha \left(\frac{{t}_{2}-{\mu }_{2}}{{\sigma }_{2}}\right)}$ refer to the selection response realized in set ${\Pi }_{1}$ and ${\Pi }_{2}$, respectively.

## Appendix 2: Maximizing selection response by optimal choice of selection thresholds

To determine the maximum of the selection response ${G}_{{\text{Tot}}}\left({t}_{1},{t}_{2},{\varvec{\xi}},{b}_{1},{b}_{2}\right)$ as a function of the thresholds ${t}_{1}$ and ${t}_{2}$, we use a Lagrange multiplier approach. We start with some basic properties of the normal distribution $N\left(\mathrm{0,1}\right)$ with pdf $\varphi \left(x\right)$ and cdf $\Phi \left(x\right)$. Let ${i}_{\alpha \left(x\right)}=\frac{\varphi \left(x\right)}{\alpha \left(x\right)}$ and $\alpha \left(x\right)={\int }_{x}^{\infty }\varphi \left(z\right)dz=1- \Phi \left(x\right)$ be the selection intensity and selected proportion, respectively, then we have $\frac{\partial \varphi (x)}{\partial x}=-x\varphi \left(x\right)$ and $\frac{\partial \alpha (x)}{\partial x}=-\varphi \left(x\right)$. Hence, setting $x=\frac{t-\mu }{\sigma }$ , we obtain.

23 $$\frac{\partial \varphi \left(\frac{t-\mu }{\sigma }\right)}{\partial t}=-\left(\frac{t-\mu }{\sigma }\right)\varphi \left(\frac{t-\mu }{\sigma }\right)\frac{1}{\sigma }.$$

24 $$\frac{\partial \alpha \left(\frac{t-\mu }{\sigma }\right)}{\partial t}=-\varphi \left(\frac{t-\mu }{\sigma }\right)\frac{1}{\sigma }.$$

Defining for given values of ${\varvec{\xi}},{b}_{1},{b}_{2}$ and ${\alpha }_{{\text{T}}}\in \left(\mathrm{0,1}\right)$:

$$\beta_{1} \left( {t_{1} } \right) = \alpha \left( {\frac{{t_{1} - \mu_{1} }}{{\sigma_{1} }}} \right) \pi_{1} ,\;\beta_{2} \left( {t_{2} } \right) = \alpha \left( {\frac{{t_{2} - \mu_{2} }}{{\sigma_{2} }}} \right) \pi_{2} ,\;g\left( {t_{1} ,t_{2} } \right) = { }\beta_{1} \left( {t_{1} } \right) + \beta_{2} \left( {t_{2} } \right) - \alpha_{{\text{T}}}$$

and

$$\begin{aligned} f\left( {t_{1} ,t_{2} } \right) &= \Delta G_{1} \left( {\left( {t_{1} - \mu_{1} } \right),\sigma_{1} ,b_{1} } \right){ }\beta_{1} \left( {t_{1} } \right) + \Delta G_{2} \left( {\left( {t_{2} - \mu_{2} } \right),\sigma_{2} ,b_{2} } \right){ }\beta_{2} \left( {t_{2} } \right) \\ & \quad + \mu_{1} \left( {{ }\beta_{1} \left( {t_{1} } \right) - \pi_{1} \alpha_{{\text{T}}} } \right) + { }\mu_{2} { }\left( {\beta_{2} \left( {t_{2} } \right) - \pi_{2} \alpha_{{\text{T}}} } \right), \\ \end{aligned}$$

we get the Lagrangian function

25 $$L\left({t}_{1},{t}_{2},\lambda \right)= f\left({t}_{1},{t}_{2}\right)+\lambda g({t}_{1},{t}_{2})$$

Thus, for given values of ${\varvec{\xi}},{b}_{1},{b}_{2}$ and $\alpha$, we obtain a necessary condition for the maximum of $\Delta {G}_{{\text{Tot}}}\left({t}_{1},{t}_{2},{\varvec{\xi}},{b}_{1},{b}_{2}\right)=$ $\frac{1}{{\alpha }_{{\text{T}}}}f\left({t}_{1},{t}_{2}\right)$ under the side condition $g\left({t}_{1},{t}_{2}\right)$ =0 by analyzing the gradient $\nabla L\left({t}_{1},{t}_{2},\lambda \right)$.

We have

$$\Delta G_{1} \left( {\left( {t_{1} - \mu_{1} } \right),\sigma_{1} ,b_{1} } \right) \beta_{1} \left( {t_{1} } \right) = b_{1} \sigma_{1} \frac{{\varphi \left( {\frac{{t_{1} - \mu_{1} }}{{\sigma_{1} }}} \right)}}{{\alpha \left( {\frac{{t_{1} - \mu_{1} }}{{\sigma_{1} }}} \right)}} \alpha \left( {\frac{{t_{1} - \mu_{1} }}{{\sigma_{1} }}} \right) \pi_{1} = b_{1} \sigma_{1} \pi_{1} \varphi \left( {\frac{{t_{1} - \mu_{1} }}{{\sigma_{1} }}} \right)$$

and

$$\Delta G_{2} \left( {\left( {t_{2} - \mu_{2} } \right),\sigma_{2} ,b_{2} } \right) \beta_{2} \left( {t_{2} } \right) = b_{2} \sigma_{2} \pi_{2} \varphi \left( {\frac{{t_{2} - \mu_{2} }}{{\sigma_{2} }}} \right).$$

Thus,

$$\frac{{\partial (\Delta G_{1} \left( {\left( {t_{1} - \mu_{1} } \right),\sigma_{1} ,b_{1} } \right) \beta_{1} \left( {t_{1} } \right)}}{{\partial t_{1} }} = - b_{1} \sigma_{1} \pi_{1} \left( {\frac{{t_{1} - \mu_{1} }}{{\sigma_{1} }}} \right)\varphi \left( {\frac{{t_{1} - \mu_{1} }}{{\sigma_{1} }}} \right)\frac{1}{{\sigma_{1} }} = - b_{1} \pi_{1} \left( {t_{1} - \mu_{1} } \right)\varphi \left( {\frac{{t_{1} - \mu_{1} }}{{\sigma_{1} }}} \right)\frac{1}{{\sigma_{1} }}$$

$$ \begin{aligned}& \frac{{\partial \Delta G_{2} \left( {\left( {t_{2} - \mu_{2} } \right),\sigma_{2} ,b_{2} } \right) \beta_{2} \left( {t_{2} } \right)}}{{\partial t_{1} }} = 0,\;\\ &\frac{{\partial \left(\mu_{1} \left( { \beta_{1} \left( {t_{1} } \right) - \pi_{1} \alpha_{{\text{Tot}}} } \right)\right)}}{{\partial t_{1} }} = - \mu_{1} \pi_{1} \varphi \left( {\frac{{t_{1} - \mu_{1} }}{{\sigma_{1} }}} \right) \frac{1}{{\sigma_{1} }},\;\\ &\frac{{\partial \left(\mu_{2} \left( { \beta_{2} \left( {t_{2} } \right) - \pi_{2} \alpha_{{\text{Tot}}} } \right)\right)}}{{\partial t_{1} }} = 0,\end{aligned} $$

$$\frac{{\partial \lambda g\left( {t_{1} ,t_{2} } \right)}}{{\partial t_{1} }} = - \lambda \pi_{1} \varphi \left( {\frac{{t_{1} - \mu_{1} }}{{\sigma_{1} }}} \right)\frac{1}{{\sigma_{1} }}.$$

Likewise,

$$\frac{{\partial (\Delta G_{1} \left( {\left( {t_{1} - \mu_{1} } \right),\sigma_{1} ,b_{1} } \right) \beta_{1} \left( {t_{1} } \right)}}{{\partial t_{2} }} = 0,\;\frac{{\partial \Delta G_{2} \left( {\left( {t_{2} - \mu_{2} } \right),\sigma_{2} ,b_{2} } \right) \beta_{2} \left( {t_{2} } \right)}}{{\partial t_{2} }} = - b_{2} \pi_{2} \left( {t_{2} - \mu_{2} } \right)\varphi \left( {\frac{{t_{2} - \mu_{2} }}{{\sigma_{2} }}} \right)\frac{1}{{\sigma_{2} }},$$

$$ \begin{aligned}& \frac{{\partial \left(\mu_{1} \left( { \beta_{1} \left( {t_{1} } \right) - \pi_{1} \alpha_{{\text{T}}} } \right)\right)}}{{\partial t_{2} }} = 0,\;\\ &\frac{{\partial \left(\mu_{2} \left( { \beta_{2} \left( {t_{2} } \right) - \pi_{2} \alpha_{{\text{T}}} } \right)\right)}}{{\partial t_{2} }} = - \mu_{2} \pi_{2} \varphi \left( {\frac{{t_{2} - \mu_{2} }}{{\sigma_{2} }}} \right) \frac{1}{{\sigma_{2} }}, \\ &\frac{{\partial \lambda g\left( {t_{1} ,t_{2} } \right)}}{{\partial t_{2} }} = - \lambda \pi_{2} \varphi \left( {\frac{{t_{2} - \mu_{2} }}{{\sigma_{2} }}} \right)\frac{1}{{\sigma_{2} }}.\end{aligned} $$

$$\frac{{\partial (\Delta G_{1} \left( {\left( {t_{1} - \mu_{1} } \right),\sigma_{1} ,b_{1} } \right) \beta_{1} \left( {t_{1} } \right)}}{\partial \lambda } = 0,\;\frac{{\partial \Delta G_{2} \left( {\left( {t_{2} - \mu_{2} } \right),\sigma_{2} ,b_{2} } \right) \beta_{2} \left( {t_{2} } \right)}}{\partial \lambda } = 0,\;\frac{{\partial (\mu_{1} \left( { \beta_{1} \left( {t_{1} } \right) - \alpha_{{\text{T}}} } \right))}}{\partial \lambda } = 0,\;\frac{{\partial (\mu_{2} \left( {\beta_{1} \left( {t_{1} } \right) - \pi_{1} \alpha_{{\text{T}}} } \right))}}{\partial \lambda } = 0$$

and

$$\frac{{\partial { }\lambda { }g\left( {t_{1} ,t_{2} } \right)}}{\partial \lambda } = g\left( {t_{1} ,t_{2} } \right)$$

Thus, we get

26 $$\begin{aligned} \frac{{\partial L\left( {t_{1} ,t_{2} ,\lambda } \right)}}{{\partial t_{1} }} & = - b_{1} \pi_{1} \left( {t_{1} - \mu_{1} } \right)\varphi \left( {\frac{{t_{1} - \mu_{1} }}{{\sigma_{1} }}} \right)\frac{1}{{\sigma_{1} }} - \mu_{1} \pi_{1} \varphi \left( {\frac{{t_{1} - \mu_{1} }}{{\sigma_{1} }}} \right) \frac{1}{{\sigma_{1} }} - \lambda \pi_{1} \varphi \left( {\frac{{t_{1} - \mu_{1} }}{{\sigma_{1} }}} \right)\frac{1}{{\sigma_{1} }} \\ & = - \frac{{\pi_{1} }}{{\sigma_{1} }}\varphi \left( {\frac{{t_{1} - \mu_{1} }}{{\sigma_{1} }}} \right)[\left( {b_{1} \left( {t_{1} - \mu_{1} } \right) + \mu_{1} + \lambda } \right] \\ \end{aligned}$$

27 $$\frac{\partial L\left({t}_{1},{t}_{2},\lambda \right)}{\partial {t}_{2}}=- \frac{{\pi }_{2}}{{\sigma }_{2}}\varphi \left(\frac{{t}_{2}-{\mu }_{2}}{{\sigma }_{2}}\right)[({b}_{2}\left({t}_{2}-{\mu }_{2}\right)+{\mu }_{2}+\lambda ]$$

28 $$\frac{\partial L\left({t}_{1},{t}_{2}, \lambda \right)}{\partial \lambda }=g\left({t}_{1},{t}_{2}\right)$$

Setting $\nabla L\left({t}_{1},{t}_{2},\lambda \right)=\left(0, 0, 0\right)$ and using that $\frac{{\pi }_{1}}{{\sigma }_{1}}\varphi \left(\frac{{t}_{1}-{\mu }_{1}}{{\sigma }_{1}}\right)>0$ and $\frac{{\pi }_{2}}{{\sigma }_{2}}\varphi \left(\frac{{t}_{2}-{\mu }_{2}}{{\sigma }_{2}}\right)>0$, we obtain from Eqs. 26 and 27 the necessary conditions: -$\lambda ={b}_{1}\left({t}_{1}-{\mu }_{1}\right)+{\mu }_{1}$ and -$\lambda ={b}_{2}\left({t}_{2}-{\mu }_{2}\right)+{\mu }_{2}$ or equivalently ${b}_{1}\left({t}_{1}-{\mu }_{1}\right)+{\mu }_{1}={b}_{2}\left({t}_{2}-{\mu }_{2}\right)+{\mu }_{2}$. Thus, the solutions ${t}_{1}^{*},{t}_{2}^{*}$ must fulfill the following conditions:

29 $$t_{1} = \frac{{b_{2} \left( {t_{2} { } - \mu_{2} } \right) + \mu_{2} - \mu_{1} }}{{b_{1} }} + \mu_{1} \;{\text{or}}\;{\text{ equivalently}}\;t_{2} = \frac{{b_{1} \left( {t_{1} - \mu_{1} } \right) + \mu_{1} - \mu_{2} }}{{b_{2} }} + \mu_{2}$$

and

30 $$\Phi \left(\frac{{t}_{1}-{\mu }_{1}}{{\sigma }_{1}}\right){\pi }_{1}+\Phi \left(\frac{{t}_{2}-{\mu }_{2}}{{\sigma }_{2}}\right){\pi }_{2}=1-{\alpha }_{{\text{T}}}$$

31 $${\text{For}}\;{\text{ the}}\;{\text{ special}}\;{\text{ case }}\;{\text{of}}\;{\text{ BLUPs}}, \, \;{\text{where}}\;b_{1} = b_{2} \; = {1}.0,\;{\text{ we}}\;{\text{ have}}\;t_{1}^{*} = t_{2}^{*} .$$

32 $${\text{For}}\;{\text{ BLUEs }}\;{\text{and}}\;\mu_{1} = \mu_{2} ,\;{\text{we }}\;{\text{get}} \;t_{1}^{*} - \mu_{1} = \frac{{b_{2} \left( {{ }t_{2}^{*} - \mu_{1} } \right)}}{{b_{1} }}.$$

Defining $${t}_{1}^{**}={t}_{1}^{*}-{\mu }_{1}$$ and $${t}_{2}^{**}={t}_{2}^{*}-{\mu }_{1} ,$$ Eqs. 29 and 30 are equivalent to $${t}_{1}^{**}=\frac{{b}_{2}{t}_{2}^{**}}{{b}_{1}}$$ and$$\Phi \left(\frac{{t}_{1}^{**}}{{\sigma }_{1}}\right){\pi }_{1}+\Phi \left(\frac{{t}_{2}^{**}}{{\sigma }_{2}}\right){\pi }_{2}=1-{\alpha }_{{\text{T}}}$$, which would be obtained if we assume $${\mu }_{1}=0$$. The improvement in $$\Delta {G}_{{\text{Tot}}}\left({t}_{1}^{*},{t}_{2}^{*},{\varvec{\xi}},{b}_{1},{b}_{2}\right)$$ relative to $$\Delta {G}_{{\text{Tot}}}\left({t}_{1}^{i},{t}_{2}^{i},{\varvec{\xi}},{b}_{1},{b}_{2}\right)$$, where $${t}_{1}^{i}={t}_{2}^{i}$$ such that $${\alpha }_{{\text{Tot}}}\left({t}_{1}^{i},{t}_{2}^{i},{\varvec{\xi}}\right)={\alpha }_{{\text{T}}}$$ or equivalently $$\Phi \left(\frac{{t}_{1}^{i}-{\mu }_{2}}{{\sigma }_{1}}\right){\pi }_{1}+\Phi \left(\frac{{t}_{2}^{i}-{\mu }_{2}}{{\sigma }_{2}}\right){\pi }_{2}=1-{\alpha }_{{\text{T}}} ,$$ can be expressed as the ratio $${\Psi }_{{\text{Tot}}}\left({\alpha }_{{\text{T}}},{\varvec{\xi}},{b}_{1},{b}_{2}\right)$$ defined in Eq. 10.

## Appendix 3: Maximizing the total selection response for factorials in hybrid breeding

Since the SC is expected to provide unbiased estimates for the GCA effects in each parent population, we have ${\mu }_{1}={\mu }_{2}=0$. Additionally, we assume that ${b}_{1}$ and ${b}_{2}$ are the regression coefficients of the regression of GCA effects on the SC in ${\Pi }_{1}$ and ${\Pi }_{2}$, respectively, and define ${\varvec{\theta}}$ =( ${\sigma }_{1}, {\sigma }_{2}, {b}_{1},{b}_{2})$. We select proportions ${\alpha }_{1}=\frac{\left|{\Gamma }_{1}\right|}{\left|{\Pi }_{1}\right|}$ and ${\alpha }_{2}=\frac{\left|{\Gamma }_{2}\right|}{\left|{\Pi }_{2}\right|}$ from ${\Pi }_{1}$ and ${\Pi }_{2}$, respectively, by applying corresponding thresholds ${t}_{1}={\sigma }_{1}{\Phi }^{-1}\left(1-{\alpha }_{1}\right)$ and ${t}_{2}={\sigma }_{2}{\Phi }^{-1}\left(1-{\alpha }_{2}\right)$. If the selected lines are mated in the form of a complete factorial design to produce the hybrids tested in the next step of the breeding program, the proportion of selected hybrids from the total set of possible hybrids is

33 $$\alpha_{{{\text{Hyb}}}} \left( {t_{1} ,t_{2} ,{\varvec{\theta}}} \right) = { }\alpha \left( {\frac{{t_{1} }}{{\sigma_{1} }}} \right) \times { }\alpha \left( {\frac{{t_{2} }}{{\sigma_{2} }}} \right).$$

The expected selection response in the hybrids among lines from ${\Pi }_{1}$ and ${\Pi }_{2}$, which were selected for their GCA in cross-combinations with genotypes from the other population, can be obtained using Eq. 6 as follows

34 $$\Delta G_{{\text{Hyb}}} \left( {t_{1} ,t_{2} ,{\varvec{\theta}}} \right) = \Delta G_{1} \left( {t_{1} ,\sigma_{1} ,b_{1} } \right) + \Delta G_{2} \left( {t_{2} ,\sigma_{2} ,b_{2} } \right),$$

with

35 $$\Delta {G}_{1}\left({t}_{1},{\sigma }_{1},{b}_{1}\right)={b}_{1}{\sigma }_{1}\frac{\varphi \left(\frac{{t}_{1}}{{\sigma }_{1}}\right)}{\alpha \left(\frac{{t}_{1}}{{\sigma }_{1}}\right)}={\frac{1}{ {\alpha }_{{\text{Hyb}}}\left({t}_{1},{t}_{2},{\varvec{\theta}}\right)}{b}_{1}}{\sigma }_{1}\varphi \left(\frac{{t}_{1}}{{\sigma }_{1}}\right) \alpha \left(\frac{{t}_{2}}{{\sigma }_{2}}\right)$$

and

36 $$\Delta {G}_{2}\left({t}_{2},{\sigma }_{2},{b}_{2}\right)={\frac{1}{ {\alpha }_{{\text{Hyb}}}\left({t}_{1},{t}_{2},{\varvec{\theta}}\right)}{b}_{2}}{\sigma }_{2}\varphi \left(\frac{{t}_{2}}{{\sigma }_{2}}\right) \alpha \left(\frac{{t}_{1}}{{\sigma }_{1}}\right)$$

Defining for given values of ${\varvec{\theta}}$ and ${\alpha }_{{\text{H}}}\in \left(\mathrm{0,1}\right)$:

$$\beta_{1} \left( {t_{1} } \right) = \alpha \left( {\frac{{t_{1} }}{{\sigma_{1} }}} \right),\;\beta_{2} \left( {t_{2} } \right) = \alpha \left( {\frac{{t_{2} }}{{\sigma_{2} }}} \right),\;g\left( {t_{1} ,t_{2} } \right) = { }\beta_{1} \left( {t_{1} } \right){ }\beta_{2} \left( {t_{2} } \right) - \alpha_{{\text{H}}}$$

and

$$f\left({t}_{1},{t}_{2}\right)={b}_{1}{\sigma }_{1}\varphi \left(\frac{{t}_{1}}{{\sigma }_{1}}\right) \alpha \left(\frac{{t}_{2}}{{\sigma }_{2}}\right) +{b}_{2}{\sigma }_{2}\varphi \left(\frac{{t}_{2}}{{\sigma }_{2}}\right) \alpha \left(\frac{{t}_{1}}{{\sigma }_{1}}\right),$$

We get the Lagrangian function

37 $$L\left( {t_{1} ,t_{2} ,\lambda } \right) = f\left( {t_{1} ,t_{2} } \right) + \lambda g\left( {t_{1} ,t_{2} } \right)$$

Thus, for given values of ${\varvec{\theta}}$ and ${\alpha }_{{\text{H}}}$, we obtain a necessary condition for the maximum of $\Delta {G}_{{\text{Hyb}}}\left({t}_{1},{t}_{2},{\varvec{\theta}}\right)=$ $\frac{1}{{\alpha }_{{\text{H}}}}f\left({t}_{1},{t}_{2}\right)$ under the side condition $g\left({t}_{1},{t}_{2}\right)$ =0 by analyzing the gradient $\nabla L\left({t}_{1},{t}_{2},\lambda \right)$. We have

38 $$\begin{aligned} \frac{{\partial L\left( {t_{1} ,t_{2} \lambda } \right)}}{{\partial t_{1} }} & = - b_{1} \sigma_{1} \frac{{t_{1} }}{{\sigma_{1} }}\varphi \left( {\frac{{t_{1} }}{{\sigma_{1} }}} \right)\frac{1}{{\sigma_{1} }}\alpha \left( {\frac{{t_{2} }}{{\sigma_{2} }}} \right) + b_{2} \sigma_{2} \varphi \left( {\frac{{t_{2} }}{{\sigma_{2} }}} \right) \times \left( { - \varphi \left( {\frac{{t_{1} }}{{\sigma_{1} }}} \right)\frac{1}{{\sigma_{1} }}} \right) + \lambda \left( { - \varphi \left( {\frac{{t_{1} }}{{\sigma_{1} }}} \right)\frac{1}{{\sigma_{1} }}\alpha \left( {\frac{{t_{2} }}{{\sigma_{2} }}} \right)} \right) \\ & = \frac{{ - \varphi \left( {\frac{{t_{1} }}{{\sigma_{1} }}} \right)\alpha \left( {\frac{{t_{2} }}{{\sigma_{2} }}} \right)}}{{\sigma_{1} }}\left[ {b_{1} t_{1} + b_{2} \sigma_{2} i_{{\alpha \left( {\frac{{t_{2} }}{{\sigma_{2} }}} \right)}} + \lambda } \right] \\ \end{aligned}$$

39 $$\frac{\partial L\left({t}_{1},{t}_{2}\lambda \right) }{\partial {t}_{2}}= \frac{-\varphi \left(\frac{{t}_{2}}{{\sigma }_{2}}\right)\alpha \left(\frac{{t}_{1}}{{\sigma }_{1}}\right)}{{\sigma }_{2}}\left[{b}_{1}{\sigma }_{1}{i}_{\alpha \left(\frac{{t}_{1}}{{\sigma }_{1}}\right)}+{b}_{2}{t}_{2}+\lambda \right]$$

40 $$\frac{{\partial L\left( {t_{1} ,t_{2} \lambda } \right)}}{\partial \lambda } = \alpha \left( {\frac{{t_{1} }}{{\sigma_{1} }}} \right) \alpha \left( {\frac{{t_{2} }}{{\sigma_{2} }}} \right) - \alpha_{{\text{H}}}$$

Thus, from $\left(\frac{\nabla L\left({t}_{1},{t}_{2},\lambda \right)}{\partial {t}_{1}},\frac{\nabla L\left({t}_{1},{t}_{2},\lambda \right)}{\partial {t}_{2}},\frac{\nabla L\left({t}_{1},{t}_{2},\lambda \right)}{\partial \lambda }\right)=\left(\mathrm{0,0},0\right)$, we get the necessary conditions $-\lambda ={b}_{1}{t}_{1}+{b}_{2}{\sigma }_{2} {i}_{\alpha \left(\frac{{t}_{2}}{{\sigma }_{2}}\right)}$ and $- \lambda = b_{1} \sigma _{1} i_{{\alpha \left( {\frac{{t_{1} }}{{\sigma _{1} }}} \right)}} + b_{2} t_{2}$ or equivalently

41 $${ b}_{1}{t}_{1}-{b}_{2}{t}_{2}={b}_{1}{\sigma }_{1} {i}_{\alpha \left(\frac{{t}_{1}}{{\sigma }_{1}}\right)}-{b}_{2}{\sigma }_{2} {i}_{\alpha \left(\frac{{t}_{2}}{{\sigma }_{2}}\right)}= \Delta {G}_{1}\left({t}_{1},{\sigma }_{1},{b}_{1}\right)-\Delta {G}_{1}\left({t}_{1},{\sigma }_{1},{b}_{1}\right)$$

and

42 $$\alpha \left(\frac{{t}_{1}}{{\sigma }_{1}}\right) \alpha \left(\frac{{t}_{2}}{{\sigma }_{2}}\right)={\alpha }_{{\text{H}}}$$

Numerical solutions $\left({t}_{1}^{o},{t}_{2}^{o}\right)$ for Eqs. 41 and 42 can be obtained using mathematical software such as Mathematica, from which we get ${\alpha }_{1}^{o}= \alpha \left(\frac{{t}_{1}^{o}}{{\sigma }_{1}}\right)$ and ${\alpha }_{2}^{o}= \alpha \left(\frac{{t}_{2}^{o}}{{\sigma }_{1}}\right)$ and $\Delta {G}_{{\text{Hyb}}}\left({t}_{1}^{o},{t}_{2}^{o},{\varvec{\theta}}\right)$. This maximum can be compared with the selection response $\Delta {G}_{{\text{Hyb}}}\left({t}_{1}^{e},{t}_{2}^{e},{\varvec{\theta}}\right)$ for ${t}_{1}^{e}={\sigma }_{1}{\Phi }^{-1}\left(1-\sqrt{{\alpha }_{{\text{H}}}}\right)$ and ${t}_{2}^{e}={\sigma }_{2}{\Phi }^{-1}\left(1-\sqrt{{\alpha }_{{\text{H}}}}\right)$, i.e., when an equal proportion ${\alpha }^{e}=\sqrt{{\alpha }_{{\text{H}}}}$ of lines is selected for GCA in each parent population. For selection based on BLUPs, where ${b}_{1}={b}_{2}=1$, Eq. 40 simplifies to

43 $${t}_{1}-{t}_{2}={\sigma }_{1} {i}_{\alpha \left(\frac{{t}_{1}}{{\sigma }_{1}}\right)}-{\sigma }_{2} {i}_{\alpha \left(\frac{{t}_{2}}{{\sigma }_{2}}\right)}$$

which corresponds to the difference in the selection differentials in ${\Pi }_{1}$ and ${\Pi }_{2}$.

## Appendix 4: Regression equation of true genetic values (TGVs) on their BLUPs

We consider the ordinary mixed linear model studied by Henderson (1975)

$$y = X\beta + Zu + e,$$

where $y$ is an $n \times 1$ observation vector, $X$ a known $n \times p$ matrix with full column rank $p$, $\beta$ an unknown fixed vector, and $Z$ is a known $n \times q$ matrix. $u$ and $e$ are unobservable random vectors with null means and

$${\text{var}}\left[ {\begin{array}{*{20}l} u \\ e \\ \end{array} } \right] = \left[ {\begin{array}{*{20}l} G & 0 \\ 0 & R \\ \end{array} } \right],$$

where $G$ and $R$ are both nonsingular and known matrices.

Let $\widehat{u}$ be the BLUP of $u$ calculated as described by Henderson (1975) and denote ${\Sigma }_{22}=$ ${\text{var}}(\hat{u})$ and $\Sigma _{{12}} = {\text{cov}}(u,\hat{u})$. Then, according to Henderson (1975), ${\Sigma }_{22}={\Sigma }_{12 }$ so that we have ${\Sigma }_{12}{\Sigma }_{22}^{-1}=I$, if ${\boldsymbol{\Sigma }}_{22}$ can be inverted,

In the case, where $$\left[\begin{array}{c}{\varvec{u}}\\ {\varvec{e}}\end{array}\right]$$ follows a multivariate normal distribution so that $\widehat{{\varvec{u}}}$ also follows a normal distribution, the regression function of ${\varvec{u}}$ on $\widehat{{\varvec{u}}}$ is linear (Anderson 1958, p. 29) and can be expressed as ${\boldsymbol{\Sigma }}_{12}{\boldsymbol{\Sigma }}_{22}^{-1}\widehat{{\varvec{u}}}={\varvec{I}}\widehat{{\varvec{u}}}$.
