# Supplementary material for: Optimizing selection based on BLUPs or BLUEs in multiple sets of genotypes differing in their population parameters
Source: Theor Appl Genet. 2024 Apr 15;137(5):104. doi: 10.1007/s00122-024-04592-2 (PMC11018695; doi:10.1007/s00122-024-04592-2)
Supplement: Supplementary file 1 — Supplementary file1 (DOCX 342 KB) [file 122_2024_4592_MOESM1_ESM.docx]

### Supplement for paper

###

### Optimizing selection based on BLUPs or BLUEs in multiple sets of genotypes

### differing in their population parameters

Albrecht E. Melchinger^1,2^, Rohan Fernando^3^ , Andreas J. Melchinger, Chris-Carolin Schön^1^

^1^ Plant Breeding, TUM School of Life Sciences, Technical University of Munich, 85354 Freising, Germany

^2^ Institute of Plant Breeding, Seed Science and Population Genetics, University of Hohenheim, 70599 Stuttgart, Germany.

^3^ Iowa State University, Department of Animal Science, Ames, IA 50011, USA

^4^ Unversity of Stuttgart, Department of Mathematics, 70569 Stuttgart, Germany


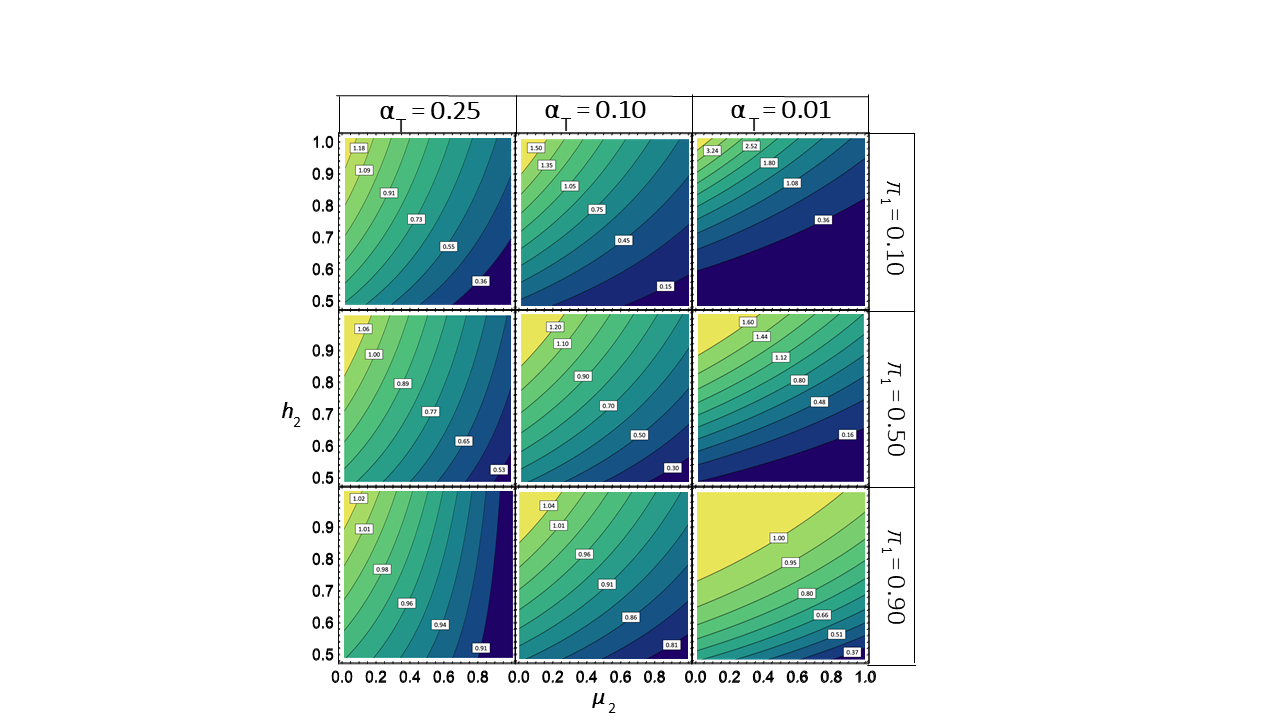
**Suppl. Figure 1S.** Contour plots for the ratio $\gamma_{1}^{i} :\pi_{1}$, indicating the shift in the proportion of genotypes from $\Pi_{1}$before ($\pi_{1})$and after ($\gamma_{1}^{i})$ truncation selection based on BLUEs, when using identical thresholds ${(t}_{1}^{i}=t_{2}^{i})$in set $\Pi_{1}$ and $\Pi_{2}$. The graphs shows $\gamma_{1}^{i} :\pi_{1}$ as a function of the mean $\mu_{2}$and $\sqrt{h_{2}^{2}}$, the square root of the heritability of the BLUEs in $\Pi_{2}$ for various values of $\pi_{1}$ and $\alpha_{T}$, the proportion of candidates selected from $\Pi_{1}\cup\Pi_{2}.$ Assumptions are $\mu_{1}$=0 and $\sigma_{u_{1}}^{2}=\sigma_{u_{2}}^{2}=1.0, h_{1}^{2}=0.50,$i.e., $\xi$ =(0, $\mu_{2},\sqrt{2}, \frac{1}{\sqrt{h_{2}^{2}}},\pi_{1}).$ The white labels attached to the contour lines show the corresponding numerical values.
